# Operando spectral imaging of the lithium ion battery’s solid-electrolyte interphase

**DOI:** 10.1126/sciadv.adg5135

**Published:** 2023-07-12

**Authors:** Jared J. Lodico, Matthew Mecklenburg, Ho Leung Chan, Yueyun Chen, Xin Yi Ling, B. C. Regan

**Affiliations:** ^1^ Department of Physics and Astronomy, University of California, Los Angeles, Los Angeles, CA 90095, USA.; ^2^ California NanoSystems Institute, University of California, Los Angeles, Los Angeles, CA 90095, USA.; ^3^ Core Center of Excellence in Nano Imaging, University of Southern California, Los Angeles, CA 90089, USA.

## Abstract

The lithium-ion battery is currently the preferred power source for applications ranging from smart phones to electric vehicles. Imaging the chemical reactions governing its function as they happen, with nanoscale spatial resolution and chemical specificity, is a long-standing open problem. Here, we demonstrate operando spectrum imaging of a Li-ion battery anode over multiple charge-discharge cycles using electron energy-loss spectroscopy (EELS) in a scanning transmission electron microscope (STEM). Using ultrathin Li-ion cells, we acquire reference EELS spectra for the various constituents of the solid-electrolyte interphase (SEI) layer and then apply these “chemical fingerprints” to high-resolution, real-space mapping of the corresponding physical structures. We observe the growth of Li and LiH dendrites in the SEI and fingerprint the SEI itself. High spatial- and spectral-resolution operando imaging of the air-sensitive liquid chemistries of the Li-ion cell opens a direct route to understanding the complex, dynamic mechanisms that affect battery safety, capacity, and lifetime.

## Teaser

Electron microscopy of an ultra-thin lithium-ion cell identifies critical side reactions as they happen during charging.

## INTRODUCTION

The lithium ion battery (LIB), already the most common rechargeable battery, also constitutes the fastest growing market segment (*1*). Given its enormous economic importance, it is perhaps surprising that basic questions remain about the chemistry governing the operation of the LIB. Ideally, charging and discharging a Li-ion cell simply moves Li^+^ ions back and forth between the cell’s graphite anode and its metal-oxide cathode as an equal number of electrons travel separately through the external circuit. However, many important and poorly understood side reactions occur as well (*2*–*6*). In particular, a fragile and multicomponent structure forms at the electrode-electrolyte interface during the first few charge-discharge cycles that plays a key role in preserving the electrode’s chemical and structural integrity over the hundreds of cycles that follow (*2*–*4*, *7*–*10*). This solid-electrolyte interphase (SEI) layer has been famously referred to as the most important and least understood component of the LIB (*3*, *11*).

In situ and operando techniques are invaluable for gaining understanding of the SEI: its structural evolution, chemical composition, and functional behavior (*12*, *13*). X-ray (*14*, *15*), electron (*13*), neutron (*16*), magnetic resonance (*17*), optical (*18*, *19*), and scanning probe (*20*–*22*) imaging and/or spectroscopy provide complementary insights into realistic Li-ion cells. Of these characterization techniques, electron microscopy, particularly transmission electron microscopy (TEM), offers a unique combination of superlative spatial resolution and spectroscopic chemical identification; traditionally, its main drawback has been the difficulties associated with applying it to the beam-sensitive, room-temperature, liquid electrolytes used in practical batteries (*12*, *13*).

High-resolution characterization of realistic lithium-ion battery (LIB) chemistries is extremely challenging (*8*, *10*, *21*, *23*–*28*). LIB sample preparation for high-resolution imaging with (scanning) TEM has previously involved invasive procedures that alter, or have the potential to alter, the structural and chemical integrity of the interface regions. Examples include freezing the sample (*29*–*31*), washing (*32*) and/or drying it (*29*, *31*–*34*), or milling it with a focused ion beam (*28*, *30*). Furthermore, with the liquid electrolytes used in most LIBs, these approaches (*29*–*31*, *33*, *34*) provide only a static snapshot of the sample. In situ imaging of LIB chemistries has been demonstrated using commercially available and homemade TEM liquid cells (*23*–*27*). However, the cells are so thick (typically ≳500 nm) that image and spectral quality, especially at core-loss energy scales, is severely degraded (*23*, *35*). Core-loss electron energy-loss spectroscopy (EELS) of lithium in liquid-cell TEM has been described as “practically impossible” (*23*).

## RESULTS

**Fig. 1.**
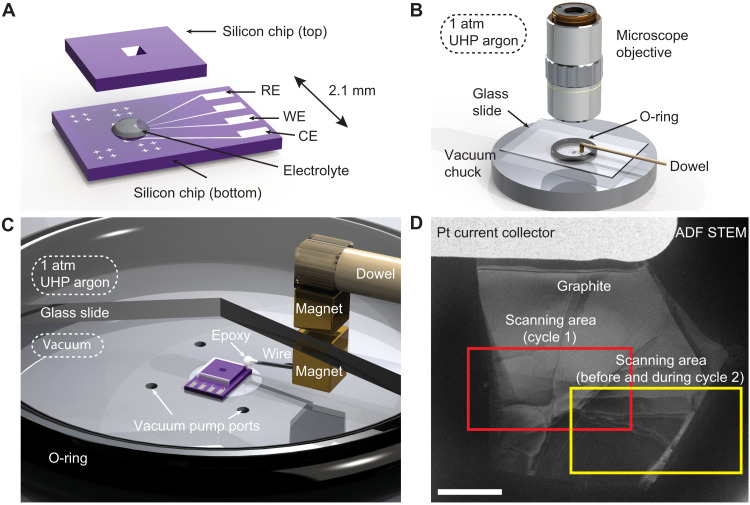
Liquid cell imaging platform for STEM EELS. (**A**) Schematic showing an exploded view of the fluid cell. A droplet of 1 M LiClO_4_ in EC:DMC serves as the electrolyte and as the lithium source (fig. S2). The top and bottom chips are epoxied together on three sides in a glove bag under 1 atm of ultrahigh-purity argon. (**B**) The fourth and final edge is sealed inside a vacuum chamber assembled inside the glove bag (not shown). (**C**) A zoom view with a cutaway in the glass slide shows how opposing NdFeB magnets provide mechanical access for epoxying inside the vacuum chamber (fig. S1). (**D**) ADF STEM image of a pristine graphite flake contacted by a platinum electrode within a sealed fluid cell. Red and yellow boxes outline the scan areas for the first and second lithiations, respectively. Scale bar, 2 μm. See fig. S13 for more details.

Using first an argon atmosphere and then vacuum to avoid air exposure, we assemble ultrathin, sealed electrochemical fluid cells that allow high-quality STEM EELS imaging of realistic Li-ion battery chemistries under operando conditions. Two silicon chips framing 20-nm-thick Si_3_N_4_ windows form the body of the TEM-compatible fluid cell (Fig. 1A). The bottom chip is instrumented with four platinum leads.

To assemble the cell, a single-crystal flake of natural graphite (*1*, *36*), which serves as the anode (the negative electrode), is first mechanically exfoliated from bulk graphite and fixed to one of the platinum leads (*37*). A ≲10-pl droplet of 1 M LiClO_4_ in ethylene carbonate:dimethyl carbonate (EC:DMC) is then deposited on the graphite electrode in an argon atmosphere, which protects the air-sensitive electrolyte (*36*, *38*). The top chip is aligned and sealed to the bottom chip with epoxy, first under 1 atm of argon and then finally under rough vacuum (Fig. 1, B and C, and fig. S1), which maintains the moisture-free environment while minimizing the pressure differential that develops across the membrane windows when the fluid cell is placed in the electron microscope’s high vacuum. If the fluid cell is sufficiently clean and thin, surface tension brings the membrane windows together during assembly, making them concave-in. Sealing the fluid cell under vacuum allows the cell to remain concave-in in the microscope’s high vacuum, unlike commercial setups, which are generally concave-out (*39*). The final vacuum-assembly step, which is directly analogous to the final vacuum-sealing step in commercial lithium-ion pouch manufacturing, is essential to achieving a thin (≲50 nm thick) liquid layer and correspondingly good STEM imaging conditions (figs. S4 and S5).

To examine the SEI formation, we bias a fluid cell in situ through two lithiations (i.e., charging half-cycles) while acquiring EELS spectrum images of the graphite-electrolyte interface. During the first lithiation, we scan an area (red box in Fig. 1D) containing the left edge of the graphite flake. The electron beam rasters from left to right (fast scan direction) and then top to bottom (slow scan direction) across the imaged area. During the second lithiation, we similarly scan an area containing the right edge of the graphite (yellow box in Fig. 1D). The substantial overlap between the two imaged regions serves as a control, in that it reveals the extent to which imaging with the electron beam modifies the electrode. In both regions, the graphite thickness is not uniform: Beyond the easily visible “bulk edge” lies an extended region of thinner few-layer graphite (i.e., multilayer graphene) that terminates at the “true edge” (fig. S3).

**Fig. 2.**
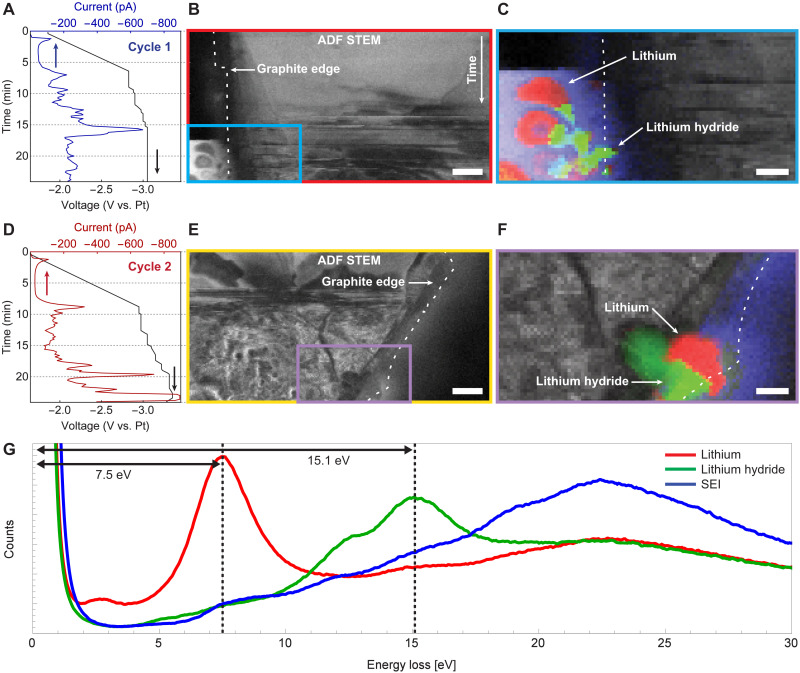
Lithiating a single-crystal graphite flake. White dashed lines indicate the graphite’s true edge (fig. S3). The measured current data from the first lithiation (**A**) are aligned and averaged so that each time point represents a single row of pixels in (**B**) the simultaneously acquired ADF STEM image. (**C**) A zoom view of the region indicated in (B), with red, green, and blue overlays on the ADF STEM image that indicate the presence of Li, LiH, and SEI, respectively, as determined by MLLS fitting (fig. S6). (**D** to **F**) Corresponding data for lithiation 2. (**G**) Low-loss EELS spectra of lithium metal (red), lithium hydride (green), and the solid-electrolyte interphase (blue). Scale bars, 500 nm (B) and (E) and 200 nm (C) and (F). See fig. S14 for more details.

Annular dark field (ADF) STEM images acquired in parallel with the EELS spectrum images provide structural information that can be related to the electrochemical transport data (Fig. 2). The transport data (Fig. 2, A and D) are oriented and scaled such that their time axis aligns with the vertical axis (i.e., the slow scan direction) of the corresponding ADF image (Fig. 2, B, C, E, and F). Lithium intercalation “events” (*37*), where variously sized groups of lithium ions are abruptly inserted between the graphene layers, give rise to electrical current pulses. These events are associated with structural changes, e.g., AB-to-AA stacking changes, in the graphite that produce contrast changes in the ADF STEM images. Because these structural reconfigurations can occur on time scales much shorter than the row-scanning time of the electron beam, the associated contrast changes often appear as horizontal stripes in the ADF STEM images (*37*).

The first cycle begins with the pristine, unlithiated graphite working electrode (WE) at its open-circuit potential of −1.86 V relative to the Pt pseudo-reference electrode. As the electrode potential is ramped at −2 mV/s, lithium ions begin intercalating the graphite, as indicated by the nonzero current (Fig. 2A). To prevent overcharging, once the current magnitude exceeds 200 pA, we switch to manual control of the electrode potential. At −3 V, a large current peak appears in the electrical transport data. Simultaneously, the graphite exhibits flake-wide ADF contrast changes (Fig. 2B) indicative of massive structural changes associated with intercalation (*37*). Shortly thereafter, several features abruptly emerge outside the graphite along the true edge (Fig. 2C).

The second lithiation is much like the first. Early in the potential ramp, the intercalation current (Fig. 2D) is small and regular. The current becomes larger and more irregular slightly before −3.0 V, and the intercalation current pulses are associated with abrupt, flake-wide contrast changes (i.e., the horizontal streaks) in the simultaneously acquired ADF STEM image (Fig. 2E). Later in the intercalation, the graphite assumes a disordered appearance in the ADF STEM image that is qualitatively different from that seen in the first lithiation. This difference between the first and the second lithiations mirrors results obtained with optical microscopy: The first lithiation irreversibly changes the graphite so as to make subsequent intercalations much more disorderly (*36*).

The EELS spectrum images add, literally, a whole new dimension to the picture provided by the transport data and the ADF STEM imaging. Converting the spectrum images to a time series of energy-filtered images (movie S1), we see distinct areas light up in various energy windows; the features grown off the graphite’s true edge are not as chemically homogeneous as ADF imaging alone would indicate. (Because the movie format conflates energy and time, the spectrum image movies S1 and S4 are best viewed in a player with a progress bar that can be dragged to specific energies.) Based on their low-loss spectral signatures, we identify two of the most prominent constituents as lithium (*25*, *30*, *40*, *41*) and lithium hydride (*30*). Lithium has a signature plasmon peak at 7.5 eV. Lithium hydride has a plasmon peak at 15.1 eV with a shoulder near 12.2 eV (*42*) due to the hydrogen core loss signal (*30*). Averaging over regions of interest (ROIs) with the strong characteristic signals, we define representative spectra for these materials, as well as for the un-intercalated graphite, the membranes-plus-electrolyte, and a third material that we will refer to as the SEI (Fig. 2G and fig. S6).

These representative spectra define a basis for multiple linear least squares (MLLS) fitting (*28*, *43*, *44*) of the entire spectrum image. We choose MLLS because, compared to other common algorithms (*45*), it is easier to implement and interpret. Applying MLLS to the Fig. 2 dataset gives composition maps of the graphite flake as it undergoes these first two lithiations. Both lithiations show Li and LiH dendrites growing adjacent to the graphite late in the charging period (Fig. 2, C and F), and in both lithiations, the dendrite growth is preceded by similar current pulses and associated changes in the ADF contrast of the graphite flake.

**Fig. 3.**
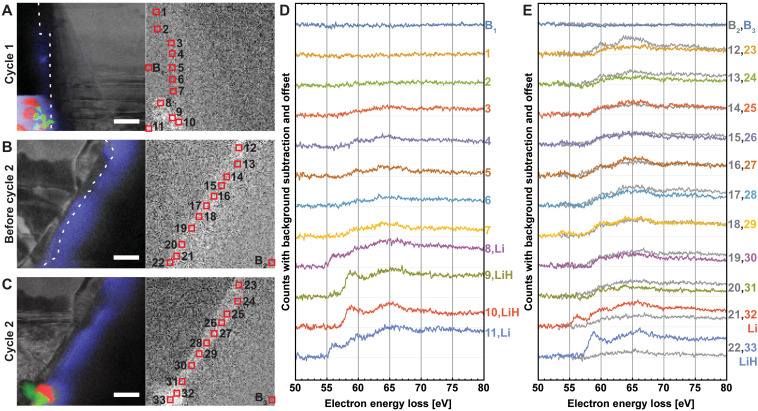
Chemical evolution at the solid-liquid interface during lithiation. ADF STEM images of the graphite flake with MLLS color overlays (see Fig. 2) (**A**) during the first lithiation, (**B**) before cycle 2, and (**C**) during lithiation 2 (left) and the corresponding energy-filtered maps of the EELS intensity integrated over 50 to 80 eV, after background subtraction (right). We emphasize that, once the reference spectra are chosen, the MLLS mapping is entirely automated over the whole field of view. (**D** and **E**) Background-subtracted spectra summed over the regions indicated by red boxes in (A) to (C). Each box is 5 × 5 pixels, which corresponds to 107 nm × 107 nm. Scale bar, 500 nm (A) to (C). See fig. S15 for more details.

To confirm the chemical identifications, we turn from the low-loss part of the spectrum to examine the Li core-loss signal (Fig. 3). During the first lithiation, as one proceeds from top-to-bottom in real space along the graphite’s edge (which also corresponds to advancing in time, as explained above), a diffuse signal in the 50- to 80-eV bandwidth is consistently increasing from zero. Spectra acquired from each of the first seven ROIs indicated on the map show quantitatively that this signal is specifically increasing near the Li K-edge at 55 eV (Fig. 3D). With the eighth ROI, this spectral signal jumps up abruptly right at the K-edge, indicating the appearance of lithium metal. The 9th and 10th ROIs also show strong Li signals, but with a chemical shift (about 2 eV) and fine structure indicative of LiH (*30*). The 11th ROI is like the 8th ROI, indicating lithium metal again. These composition identifications based on the Li core-loss signals alone (Fig. 3) are thus entirely consistent with the identifications made based on the low-loss signals alone (Fig. 2).

After the first lithiation, the graphite electrode’s potential is ramped at 2 mV/s from −3.05 V to 0 V and disconnected. The spectrum image acquired in this condition (Fig. 3B) shows a diffuse lithium core-loss signal all along the graphite edge (Fig. 3D, gray curves #12 to #22). The individual ROIs all exhibit a Li signal, but generally with a chemical shift that is perhaps larger than that of LiH. Thus, while the graphite has returned to the uncharged state, the edge of the electrode has not returned to its pristine condition. This diffuse lithium core-loss signal represents an irreversible capacity loss that originates from the SEI that forms during the first lithiation and persists thereafter (*3*, *5*–*7*), as we will return to discuss shortly.

During the second lithiation, the diffuse lithium signal in the early ROIs #23 to #31 does not develop further, indicating that the SEI is roughly unchanging. On the other hand, the late ROIs #32 and #33 show spectra indicating the appearance of Li and LiH, respectively. Again, the core-loss spectra present a consistent picture with the low-loss spectra: Both show Li and LiH dendrites appearing at the end of the lithiation, when the potential is lowest, and in the same locations.

The data of Figs. 2 and 3 are acquired with a beam current of 75 pA, a probe full-width at half maximum of 0.7 nm, and a pixel size of 21 nm (table S1). Thus, with a dwell time of 50 ms, the effective fluence [often colloquially referred to as “dose” (*46*)] per spectrum image can be quantified as 6 × 10^5^ *e*/Å^2^ or 500 *e*/Å^2^, depending on whether the relevant averaging area is taken to be the beam area or the pixel area, respectively (*46*, *47*). Although this distinction is not always made (*30*) [or the fluence is not reported at all (*25*, *33*)], the spectrum acquired in a given spectrum image reflects the damage implied by the larger fluence number.

Regardless, repeated STEM imaging with these dose conditions does not substantially affect the electrode, either in how the anode absorbs electrical charge or in how the graphite/lithium structure evolves morphologically. The transport data for the first two lithiations are very similar, and the boundaries of the thrice-imaged (Figs. 2 and 3) control area can barely be distinguished (see also the delithiation movies S2 and S3). Put another way, lithiation and delithiation—the processes under study—have a far greater effect on the graphite electrode (*36*) than the STEM spectrum imaging. Viewed together, Figs. 2 and 3 and movies S1 to S3 demonstrate that this electrochemical cell can be cycled multiple times, without appreciable degradation of the electrode due to the imaging electron beam, and with enough EELS signal to robustly locate and identify the Li and LiH species of dendrite in both low-loss and core-loss spectra. Compared to the electrode, however, the SEI is not robust in the electron beam (figs. S9 to S12 and movie S4).

**Fig. 4.**
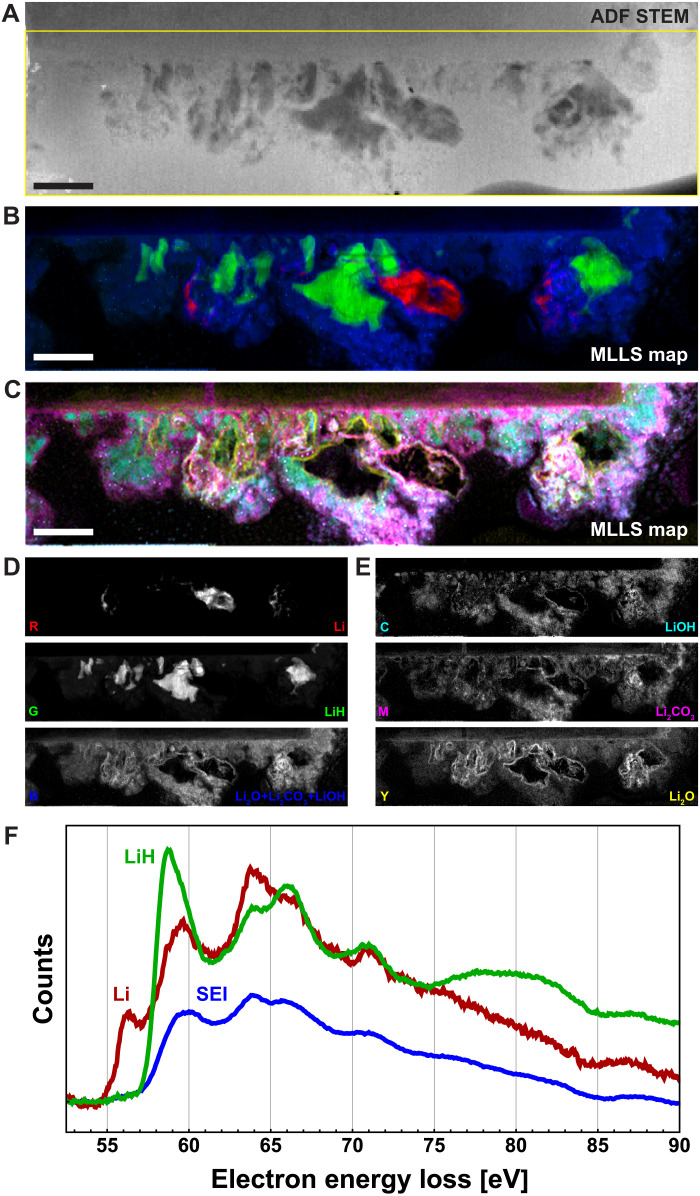
Denser, more developed SEI. (**A**) ADF STEM survey image of a graphite electrode with an SEI formed by two complete lithiation-delithiation cycles in situ. The graphite occupies a rectangular region extending from the upper left corner across 90% of the top edge. (**B**) Red, green, and blue (RGB) and (**C**) cyan, magenta, yellow (CMY) MLLS composite images (see Materials and Methods) based on a 102.4-eV bandwidth spectrum image with field of view indicated by the yellow box in (A). (**D** and **E**) Corresponding grayscale MLLS images showing the individual components of (B) and (C), respectively (fig. S7). Scale bars, 500 nm. (**F**) Background-subtracted Li core-loss signals found by calculating intensity-weighted averages over the maps (D). See fig. S16 for more details.

To acquire a more detailed spatial and spectroscopic picture of the SEI, we produce a complete SEI layer that is denser and more developed by twice ramping a second LIB cell from its open-circuit potential in the pristine state to −5.7 V and back. This aggressive cycling generates dendrites, some gas evolution, and the desired SEI. ADF STEM imaging shows a variety of dendritic features adjacent to the graphite edge (Fig. 4A), but the morphology of these features alone is insufficiently informative to allow for chemical identification (*30*). Viewed with ADF imaging (Fig. 4A), the SEI appears to be relatively uniform and barely distinguishable from the electrolyte.

Spectrum imaging in a 0- to 90-eV bandwidth (table S1) allows us to map the SEI with chemical specificity and nanoscale spatial resolution. We again apply MLLS (Fig. 4, B to E, and fig. S7), this time using seven reference spectra: four acquired from the spectrum image itself (Li, LiH, graphite, and background) and three measured previously (Li_2_O, Li_2_CO_3_, and LiOH) (*48*). The combination of EELS spectrum imaging and MLLS analysis reveals what ADF imaging does not: The layer decorating the graphite edge is chemically heterogeneous.

This layer (Fig. 4B) consists of lithium, lithium hydride, and a diffuse “SEI” (Fig. 4E) that surrounds the Li and LiH. This “extended” SEI is ∼500 nm wide and unlikely to survive any sample preparation involving washing (*30*). The Li and LiH, both rich in low-*Z* lithium, generate less scattering into the dark field than the background electrolyte and SEI, and they thus appear dark in the ADF image. Both the Li and the LiH are near but not directly adjacent to the graphite electrode. These dendrites are disconnected near their attachment points (*27*, *30*, *49*) during the delithiation process and represent partially inactive or completely inactive (i.e., “dead”) lithium that does not have a metallic connection to the electrode (*9*, *26*, *30*).

This MLLS decomposition shows lithium carbonate and lithium hydroxide with similar, but not identical, spatial distributions throughout the SEI. The combination image “Li_2_O + Li_2_CO_3_ + LiOH” (Fig. 4D), the sum of the three listed components (Fig. 4E), shows the SEI with the same spatial extent as that found by choosing a reference spectrum from a representative SEI region in the spectrum image (fig. S9). The strong, well-resolved lithium oxide signal in a thin layer surrounding the LiH and Li dendrites indicates that the dendrites have an oxide shell that is ≲10 nm thick. Of course, MLLS decomposition only reveals compounds represented in the reference spectra basis. Lithium hydroxide monohydrate and any number of lithium alkyl carbonates are likely present but unidentified, as their fingerprints are not included. Future studies would benefit greatly from the creation of an extended library of low-loss EELS reference spectra for SEI compounds.

The Li, LiH, and SEI have enough areal density to give high-quality Li core-loss spectra (Fig. 4F). These spectra, with their superior signal-to-noise ratio and well-resolved near-edge fine structure and chemical shifts (*28*–*30*, *33*), are in excellent agreement with those of Fig. 3 (D and E). Because the lithium carbonate and hydroxide signals are essentially co-located, independent core-loss spectra for these materials cannot be determined from these data. However, these ultrathin liquid cells provide such good spectroscopic access that, with a comprehensive set of reference spectra, one could apply MLLS also to the Li core-loss data (*28*) and thereby obtain time-resolved chemical identification of the SEI that is independent of, and complementary to, the low-loss fingerprinting.

## DISCUSSION

The complexity of the SEI’s composition, structure, and formation dynamics is fully evident in our data. Under operando conditions and thus without invasive sample processing, we see an SEI that ranges from tens to hundreds of nanometers thick, depending on the charging conditions (*2*, *4*, *30*, *33*). We see no remarkable differences in the structure of the SEI surrounding Li dendrites versus that surrounding LiH dendrites (*30*). The SEI that we observe has a composition that is fully consistent with that proposed by the well-known “mosaic” model (*4*, *6*). On the other hand, excepting the dendrites and their oxide shells (which are not necessary nor desirable components of an SEI), we see no structures that can fairly be described as being akin to the tiles of a mosaic. Although, for instance, the relative concentrations of Li_2_O and Li_2_CO_3_ vary throughout the SEI, with our nanometer-scale spatial resolution, we see no evidence for abrupt “grain boundaries” between microphases (*5*, *6*). Pressed to give an analogy, we would say that the SEI appears to be more like pastry dough, where incomplete mixing of flour, water, salt, sugar, and fat produces a structure that is not homogeneous, but not as well segregated as a mosaic either.

In conclusion, we observe a chemically heterogeneous SEI that consists primarily of inorganic Li compounds, as has been seen previously using other methods (*4*–*6*). However, operando STEM EELS allows this interfacial layer to be observed, as it grows, with a combination of spatial resolution and chemical identification that is unmatched by other techniques. We clearly see, for instance, not only Li and LiH dendrites but also their nanometer-scale oxide shells. Unfortunately, however, here we are only able to detect and map the SEI compounds that qualify as well-known suspects. This shortcoming is not fundamental. Expanded libraries of EELS fingerprints would allow these methods to provide a more complete picture of the form and function of the SEI, without adding experimental complexity.

## MATERIALS AND METHODS

### Fluid cell fabrication

Silicon sample-biasing chips with 20-nm-thick, 15 μm × 70 μm electron-transparent Si_3_N_4_ windows and instrumented with Ti/Pt (5/25 nm) electrodes are microfabricated as described previously (Fig. 1A) (*37*). Flakes of natural graphite (NGS Naturagraphit GmBH) are mechanically exfoliated from bulk with adhesive tape and stamped onto a sacrificial Si/SiO_2_ (500 μm/80 nm) wafer. Under an optical microscope, a flake with thickness in the desired 15- to 40-nm range is identified. Using the wet transfer method, this flake is then deposited on an electrode on a “bottom” sample-biasing chip (*37*).

To prevent electrochemistry on the Ti/Pt electrode from obscuring the graphite’s electrochemical contribution to the electrical current, the entire chip is blanketed with a 20-nm conformal layer of aluminum oxide (Al_2_O_3_) via atomic layer deposition (ALD). Using a photoresist mask patterned with optical lithography and a buffered oxide etch (BOE), unwanted Al_2_O_3_ is removed from the contact pads outside the cell and from the graphite, the counter electrode (CE), and the reference electrode (RE).

The fluid cells are assembled and sealed at room temperature in a custom-built argon atmosphere glove bag. A ∼0.2-pl droplet of 1 M lithium perchlorate (LiClO_4_) in EC [(CH_2_O)_2_CO]:DMC [OC(OCH_3_)_2_] (v:v = 1:1) acts as the electrolyte and the lithium source. Color changes in a graphite flake (fig. S2) demonstrate that lithium intercalates the graphite. These color changes (*36*) confirm that the electrolyte contains sufficient lithium to fully intercalate flakes of the sizes used in this study.

A second “top” chip with a matching electron transparent Si_3_N_4_ window seals the fluid cell. Using micromanipulators, the top chip is manually maneuvered above the bottom chip with the graphite, electrodes, and electrolyte, until the electron-transparent windows of two chips are aligned. Three of the four edges are sealed under one atmosphere of argon with vacuum-compatible epoxy. To minimize the pressure differential present across the Si_3_N_4_ membranes when the cell is in the TEM, the fourth and final edge is sealed under house vacuum using the same epoxy (Fig. 1, B and C, and fig. S1). The thickness of the cells (figs. S4 and S5) is determined using EELS and the “log-ratio” method (*50*).

### STEM spectrum imaging and in situ biasing

A Hummingbird Scientific biasing holder and a Gamry 600 potentiostat are used to electrically drive the fluid cells in situ while imaging them with STEM. All STEM images are acquired in a JEOL JEM-2100F at 200-kV accelerating voltage with an 80-mm STEM camera length. EELS spectra are acquired with a Gatan Quantum 963 spectrometer with a STEM beam convergence angle α = 9 mrad and a spectrometer collection angle β = 6 mrad through a 2.5-mm aperture (or 12 mrad through a 5-mm aperture). We achieve a zero-loss peak (ZLP) with a full-width at half-maximum of 0.75 eV. Additional imaging parameters are given in table S1. Buffered current and voltage output signals from the potentiostat are digitized and recorded synchronously with the STEM data, allowing for line-by-line correlation between the images and the electrical transport data.

### MLLS fitting

To map the different chemical components (e.g., lithium, lithium hydride, graphite, and SEI) in the electrochemical fluid cells, multiple linear least-square (MLLS) regression is used to decompose the EELS spectrum at each beam position (*28*, *43*, *44*). For each material, a representative (spatial) region of interest (ROI) is located in the spectrum image that is structurally and spectrally homogeneous. The EELS spectra inside this ROI are summed and then normalized such that the total intensity of the ZLP is unity. This normalized average spectrum is used as a reference spectrum for the MLLS fitting. In MLLS fitting, the EELS spectrum at each beam position is decomposed into a best-fit linear combination of the reference spectra over a particular energy range. (“Best fit” is defined as the list of amplitudes that minimizes the squared deviations between the spectral decomposition and the actual spectrum.) We fit over 4 to 40 eV for Figs. 2 and 3 and over 5 to 25 eV for Fig. 4, with the range limited in the second case by the data available in (*48*). The EELS spectra to be fit are normalized relative to their ZLP in the same way as the reference spectra are, and the spectral amplitudes are constrained to be nonnegative. The constraint is enforced by an iterative fit algorithm in Mathematica, which, if a negative amplitude is detected, sets the amplitude of the corresponding reference spectrum to zero and refits until all of the best-fit amplitudes are nonnegative. Ideally, the best-fit amplitudes are then indicative of the amount of the corresponding material present at that STEM beam position. Repeating the MLLS procedure at each beam position thus generates a distribution map for each material.

Color coding and overlaying the MLLS distribution maps can produce a quantitative map showing how the different chemical constituents (e.g., lithium, lithium hydride, and SEI) are distributed relative to one another across the field of view. To create an RGB (red, green, and blue) composite image of three SEI components, grayscale images of the individual SEI components are converted to red, blue, and green intensities and simply added. To create a CMY (cyan, magenta, yellow) composite image of three SEI components, the grayscale images of the individual components are converted to cyan, magenta, and yellow images first. (Equal amounts of green and blue RGB value, red and blue RGB value, and red and green RGB value give rise to the cyan, magenta, and yellow colors, respectively.) These individual images are then added together to produce a CMY composite image, which, unlike an RGB composite image, might be saturated in some areas. Despite this defect, we use CMY for Fig. 4C to avoid indicating different chemicals with the same color, and because the colors in RGB can be difficult to distinguish (RG) or see clearly on a black background (B).
