# Supplementary material for: Operando spectral imaging of the lithium ion battery’s solid-electrolyte interphase
Source: Sci Adv. 2023 Jul 12;9(28):eadg5135. doi: 10.1126/sciadv.adg5135 (PMC10337899; doi:10.1126/sciadv.adg5135)
Supplement: Supplementary file 1 — Table S1 Legends for movies S1 to S4 Figs. S1 to S16 [file sciadv.adg5135_sm.pdf]

Science Advances
AAAS

Supplementary Materials for

Operando spectral imaging of the lithium ion battery's solid-electrolyte interphase

Jared J. Lodico *et al.*

Corresponding author: B. C. Regan, regan@physics.ucla.edu

Sci. Adv. **9**, eadg5135 (2023)
DOI: 10.1126/sciadv.adg5135

The PDF file includes:

Table S1
Legends for movies S1 to S4
Figs. S1 to S16

Other Supplementary Material for this manuscript includes the following:

Movies S1 to S4

SM

SM.1 Imaging Parameters Summary

	Figs. 2–3		Fig. 4		
	surveying	low-loss	surveying	low-loss	core-loss
beam current (pA)	75	75	300	300	300
dwell time (ms)	0.019	48	0.19	35	100
pixel size (nm)	9.8	21	16	11.1	30.3
probe size (nm)	0.7	0.7	1.5	1.5	1.5
pixel/probe area ratio	250	1200	150	70	520
pixel fluence ($e/\text{Å}^2$)	1	490	14	5300	2000
probe fluence ($e/\text{Å}^2$)	230	580,000	2000	370,000	1,100,000
image size (pixels)	1024×1024	240×120	512×512	125×511	50×50
dispersion (eV/bin)	n.a.	0.05	n.a.	0.05	1
full bandwidth (eV)	n.a.	102.4	n.a.	102.4	11 to 2059
vertical binning	n.a.	5	n.a.	10	1
GIF aperture (mm)	2.5	2.5	2.5	2.5	5

Table S1: Imaging parameters. To ensure that the 'low-loss' spectra capture the entire zero-loss peak, we acquire an energy range of roughly -10 to 90 eV in those datasets. We write 'low-loss' because these spectra also capture the Li core-loss signal near 55 eV, which is at a particularly low energy due to lithium's small atomic number $Z = 3$ [42]. The 'core-loss' spectrum image that is acquired after the Fig. 4 low-loss spectrum image spans 11–2059 eV, which covers the carbon, nitrogen, and oxygen K-edges. This spectrum image is discussed in Figs S9–S12.

SM.2 Movie captions

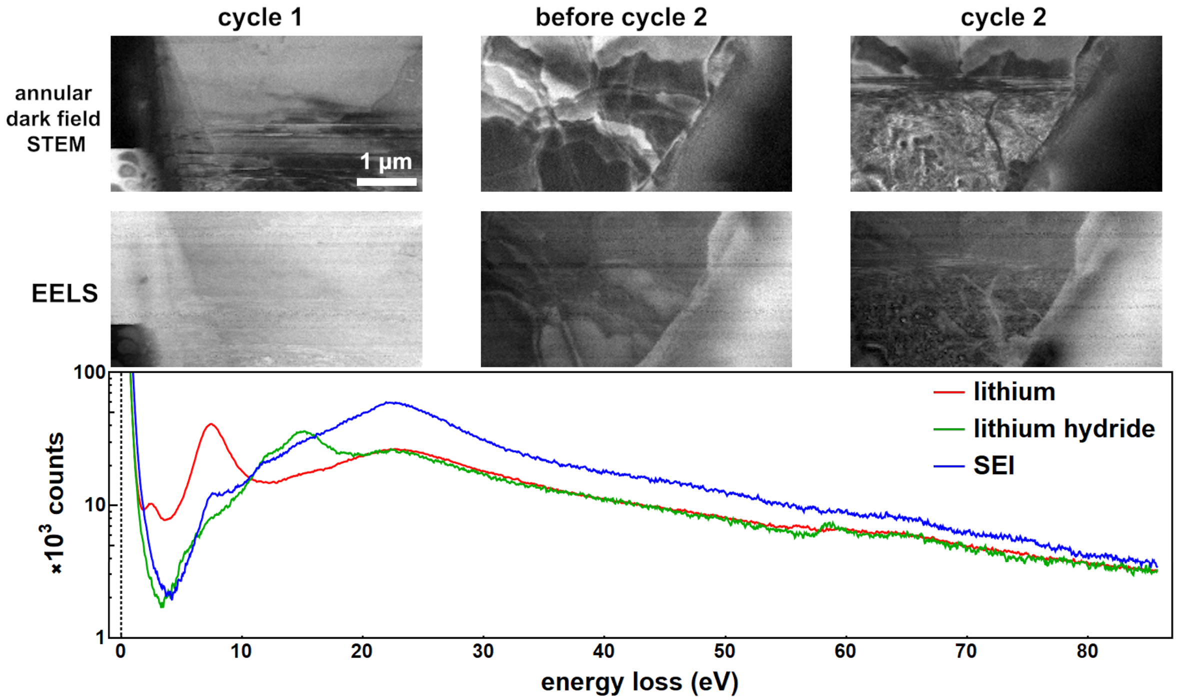

Movie S1: **Spectrum images of first lithiation, delithiated graphite, and second lithiation.** This movie combines the data from the three spectrum images that are featured in Fig. 2 and Fig. 3. ADF images (top row), energy filtered images (second row, 50 meV bandwidth), and the full spectra for several reference materials (bottom, see Fig. 2) are shown together. The particular energy slice shown in the energy filtered image is indicated by the dotted line on the bottom plot. The frame shown in the still figure above this caption highlights the LiH signal, which appears strongly in dendrites in the lower left of the cycle 1 field of view and in the lower right of the cycle 2 field of view. To best explore the data set, a movie player with a status bar that can be dragged to highlight various energy windows of interest is recommended for viewing.

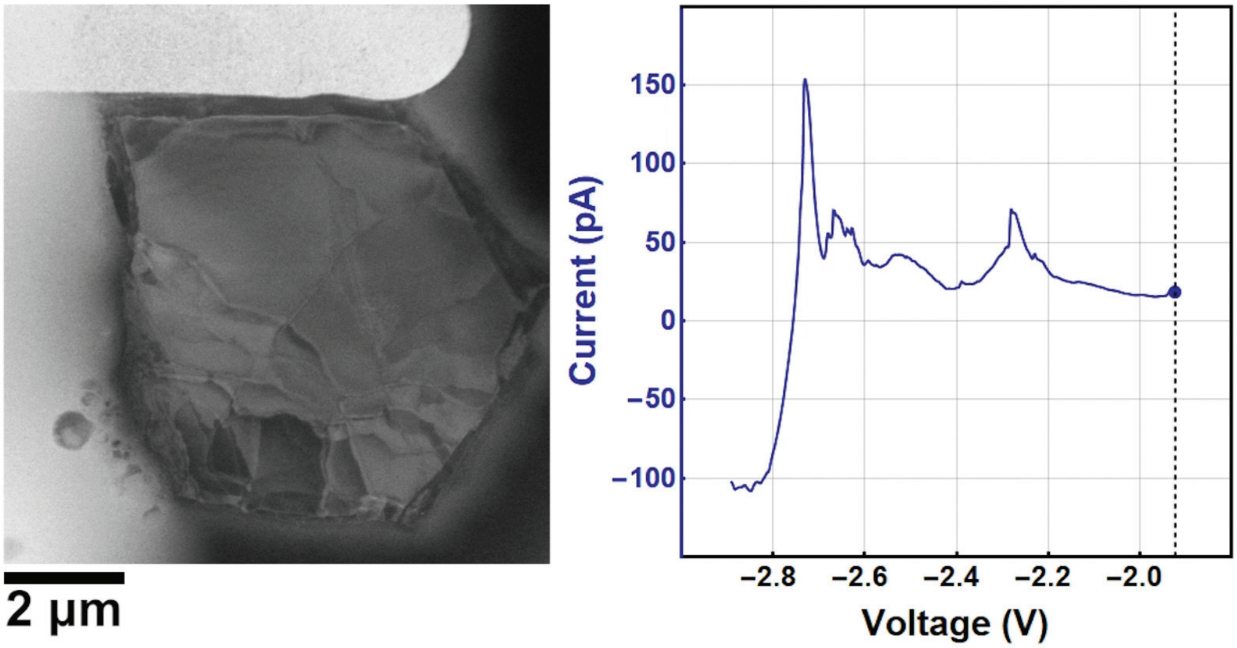

Movie S2: **Annular dark field imaging of first delithiation.** ADF STEM images of a graphite flake (left) in the process of delithiating are synchronized with the cyclic voltammogram (CV) (right). Here the potential is ramped at a constant rate of 2 mV/s. The 1024 × 1024 pixel ADF STEM images that constitute the movie are acquired with manual timing at an approximate rate of one frame per 30 s, where the frame acquisition time is 20 s and the time required to save each image is approximately 10 s. The video plays the 15 ADF images at a rate of 1 frame per second. To see the relatively small effect of imaging vs. lithiation/delithiation on this sample, see also Fig. 1 and Movie S3.

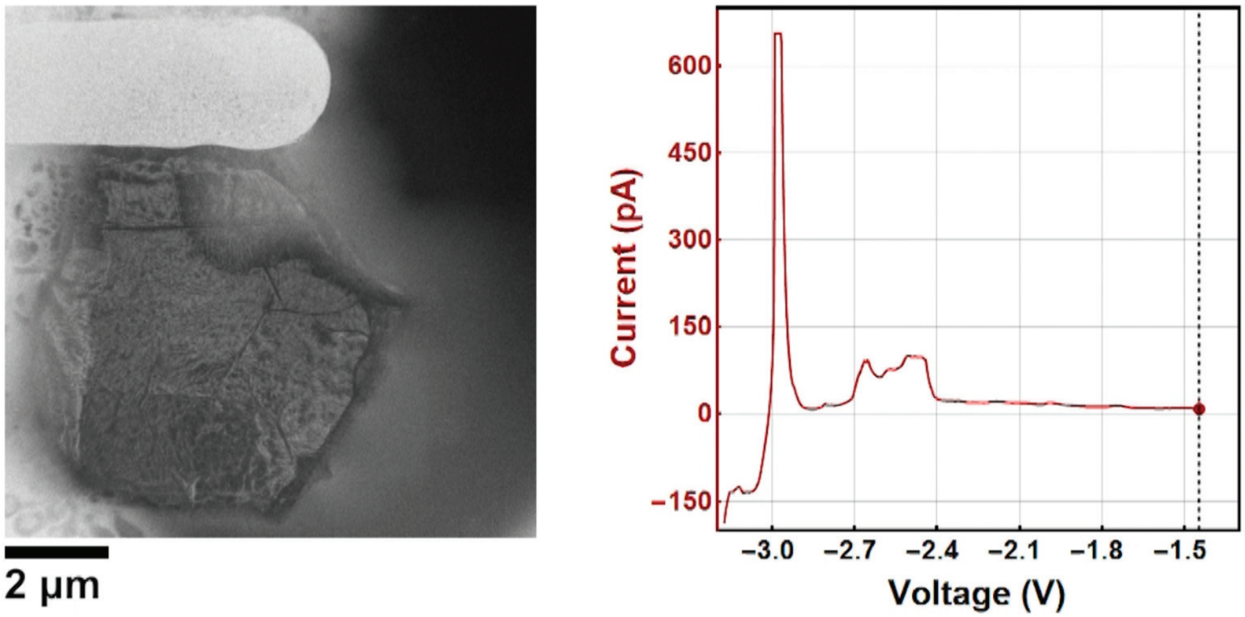

Movie S3: **Annular dark field imaging of second delithiation.** ADF STEM images of a graphite flake (left) in the process of delithiating are synchronized with the cyclic voltammogram (CV) (right). Here the potential is ramped at a constant rate of 2 mV/s. The first seventeen 1024×1024 pixel ADF STEM images (21 images in total constitute the movie) are acquired with manual timing at an approximate rate of one frame per 30 s, where the frame acquisition time is 20 s and the time required to save each image is approximately 10 s. The delays between the last four ADF images are longer (up to several minutes). To see the relatively small effect of imaging vs. lithiation/delithiation on this sample, see also Fig. 1 and Movie S2.

In this movie, and perhaps in Movie S2, some motion of the electrolyte is evident. This motion has little effect on the measured current (see particularly the time $t > 10$ s in this movie), which highlights another advantage of capping the electrodes with ALD alumina. The working electrode is immersed where it is bare, except perhaps over some small regions of graphite shown (the Pt current collector for the working electrode is covered with ALD alumina). Thus, electrolyte motion is not producing sizable electrical currents. The measured electrical currents are due to chemistry (intercalation, dendrite growth, SEI formation, etc.), as desired.

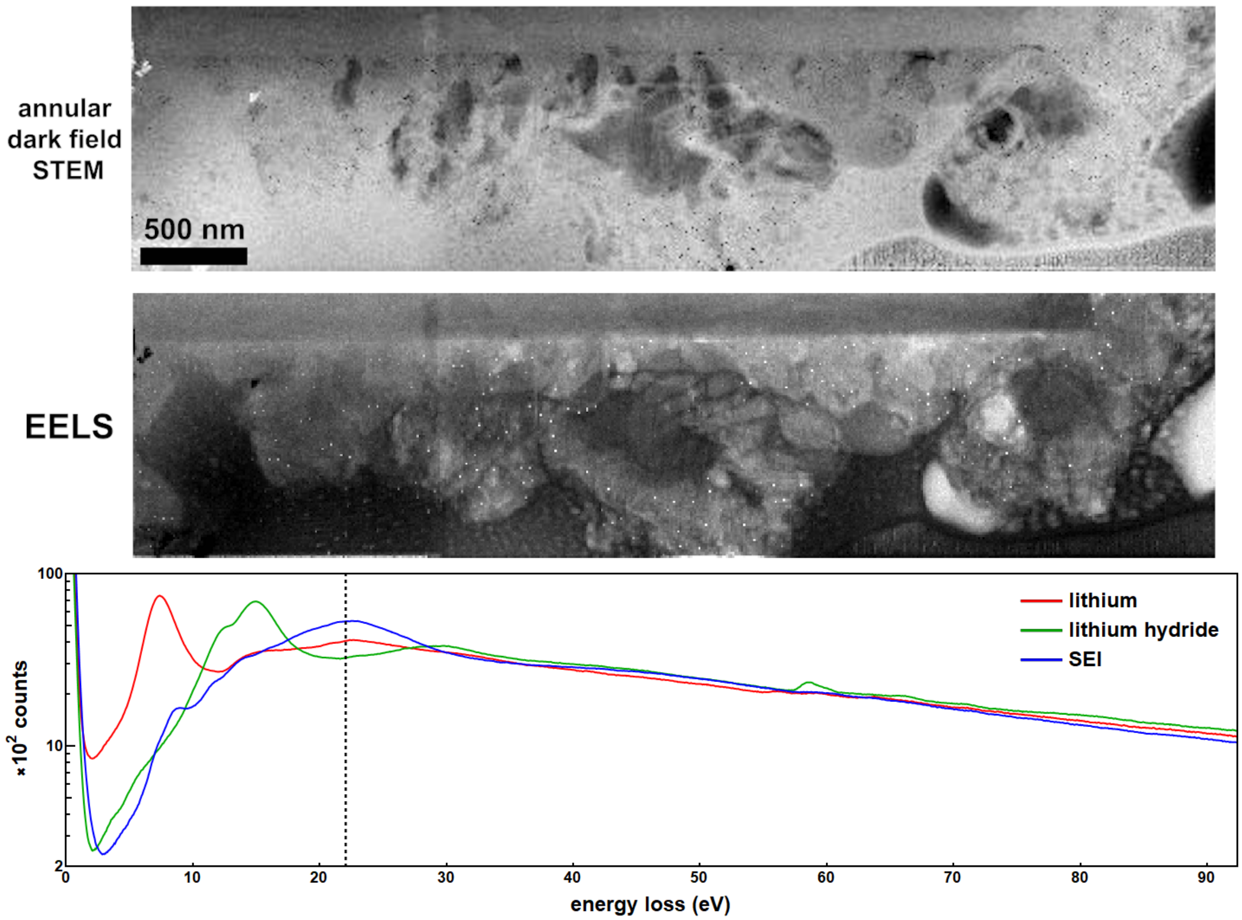

Movie S4: **Spectrum image of well-developed SEI.** This movie summarizes the spectrum image used to generate Figs. 4B–E and Fig. S8. The ADF STEM image shown on top is acquired simultaneously with the spectrum image. As with Movie S1, the scanning dotted line indicates the 50 meV energy slice shown in the EELS image. (Again, a movie player with a status bar that can be dragged to highlight various energy windows of interest is recommended for viewing.) The ADF image shows some changes from the previously acquired survey image (Fig. 4A). For example, a dark region at the far right, most likely a gas bubble, has evolved. Other signs of beam damage include the single-pixel-size spots in the spectrum image that are brighter than their surroundings at low energy and darker at high energy. We attribute these spots to chance overlap between the exact beam position and a location damaged by the beam previously (recall that the probe size is much smaller than the pixel size — see Table S1) during survey imaging. These spots appear only in the SEI and appear more frequently with repeated imaging, highlighting the particular sensitivity of the SEI to beam damage.

SM.3 Supplementary Figures

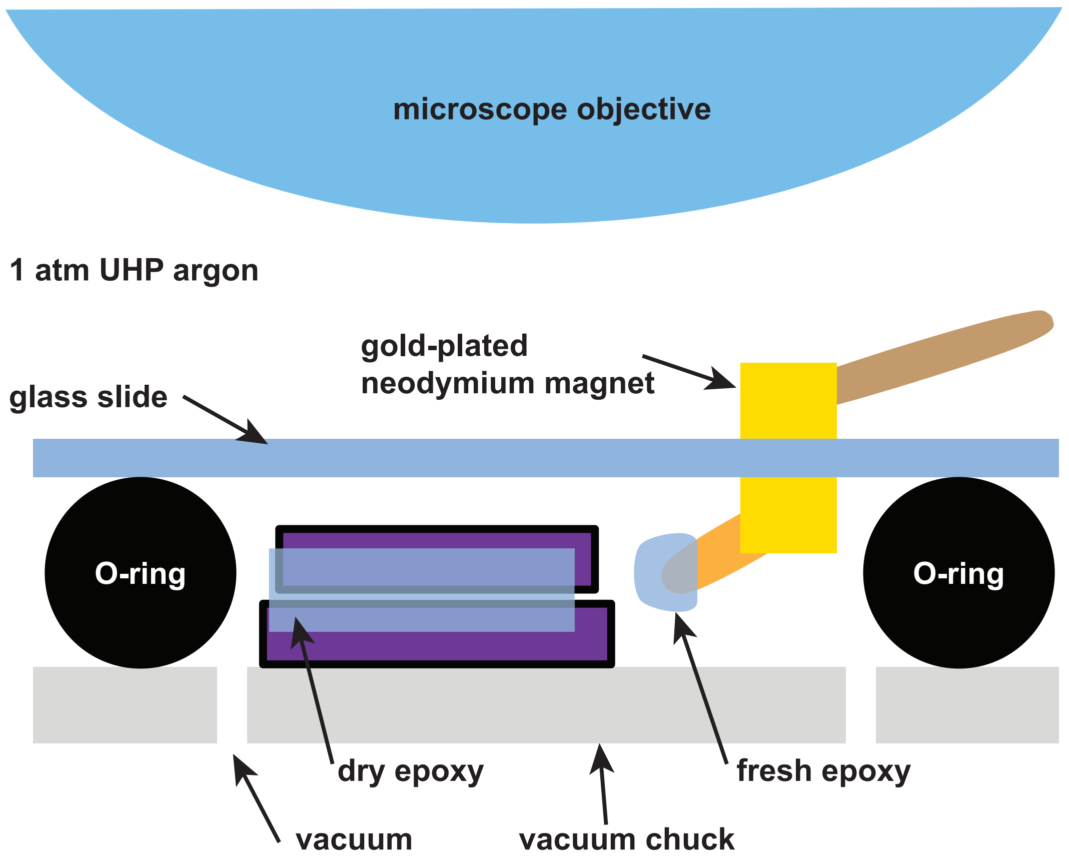

Figure S1: **Fluid cell assembly diagram.** The final steps of the cell assembly take place in a custom-made glove bag under an argon atmosphere. Inside the bag, three sides of the cell are epoxied together using the micromanipulators in a probe station equipped with an optical microscope. The fourth and final side is epoxied under vacuum (see Fig. 1 and Methods). The small, easily assembled vacuum chamber sits under the microscope objective and consists of a custom vacuum chuck, an O-ring, and a glass slide as shown (not to scale). Mechanical access for applying the epoxy is provided by a pair of 2-mm cube NdFeB magnets that transmit forces applied to the magnet outside the chamber, through the glass, to the magnet inside the chamber. The magnets slide freely on the glass, allowing the operator to apply the final, sealing coat of epoxy under vacuum, and thus to create a fluid cell that will remain thin in the TEM vacuum.

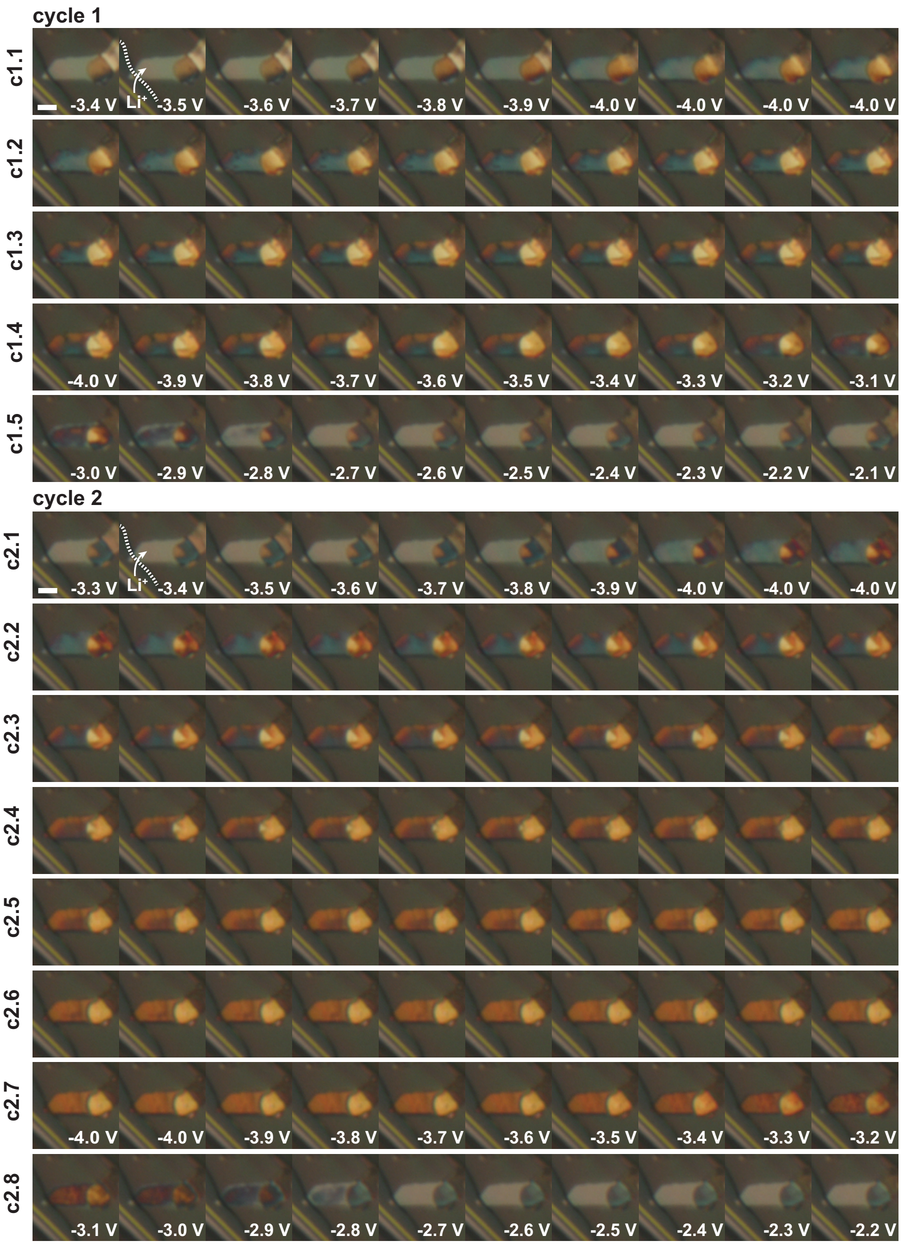

Figure S2: **Optical view of graphite lithiation/delithiation in a TEM fluid cell.** A TEM fluid cell, assembled as described in Methods, is imaged optically [36] over two sequential lithiation/delithiation cycles. The image acquisition times are separated by 10 s, so each row represents a a time span of 100 s. The potential ramp rates are ± 10 mV/s between the open circuit potential (OCP) of −0.5 V vs. Pt and −4.0 V vs. Pt, and the potential is held at −4.0 V for 250 s and 540 s after the first and second lithiations, respectively. The scale bars shown are 3 μm. The graphite's color changes [36] from gray to gold and back demonstrate full lithiation (to LiC_6) and delithiation in the TEM electrochemical fluid cell. Two frames with Li^+ labels highlight the boundary of the electrolyte; the left tip of the flake is immersed, while the remainder on the right is dry.

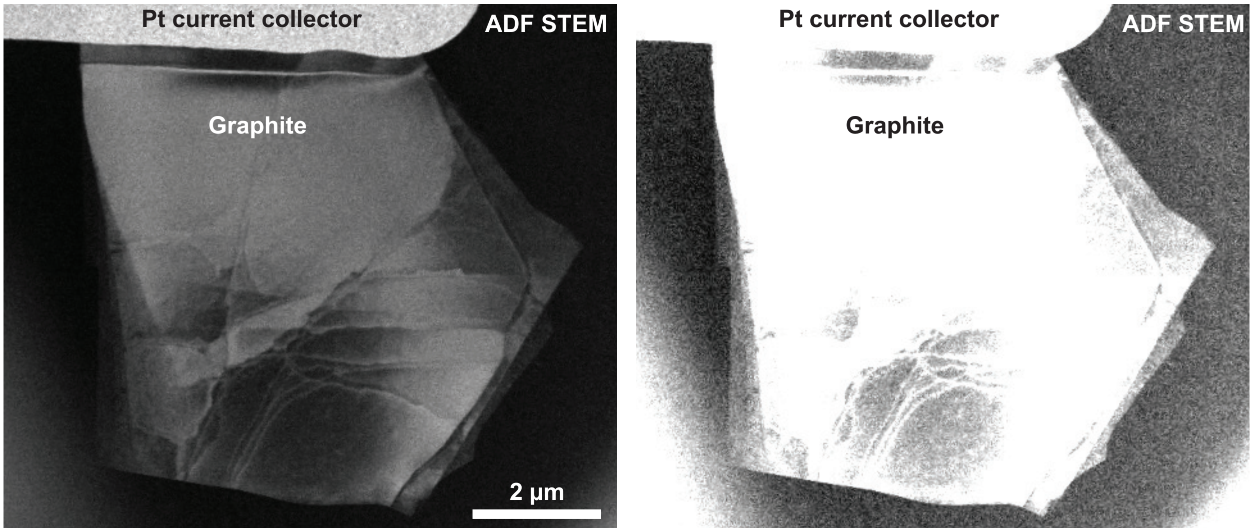

Figure S3: **Graphite edge determination.** (left) Lower magnification ADF STEM image of the graphite flake in Figs. 1, 2, and 3, shown with typical contrast and brightness values. (right) Adjusting the contrast and brightness of the left image makes the true edges of the graphite flake visible.

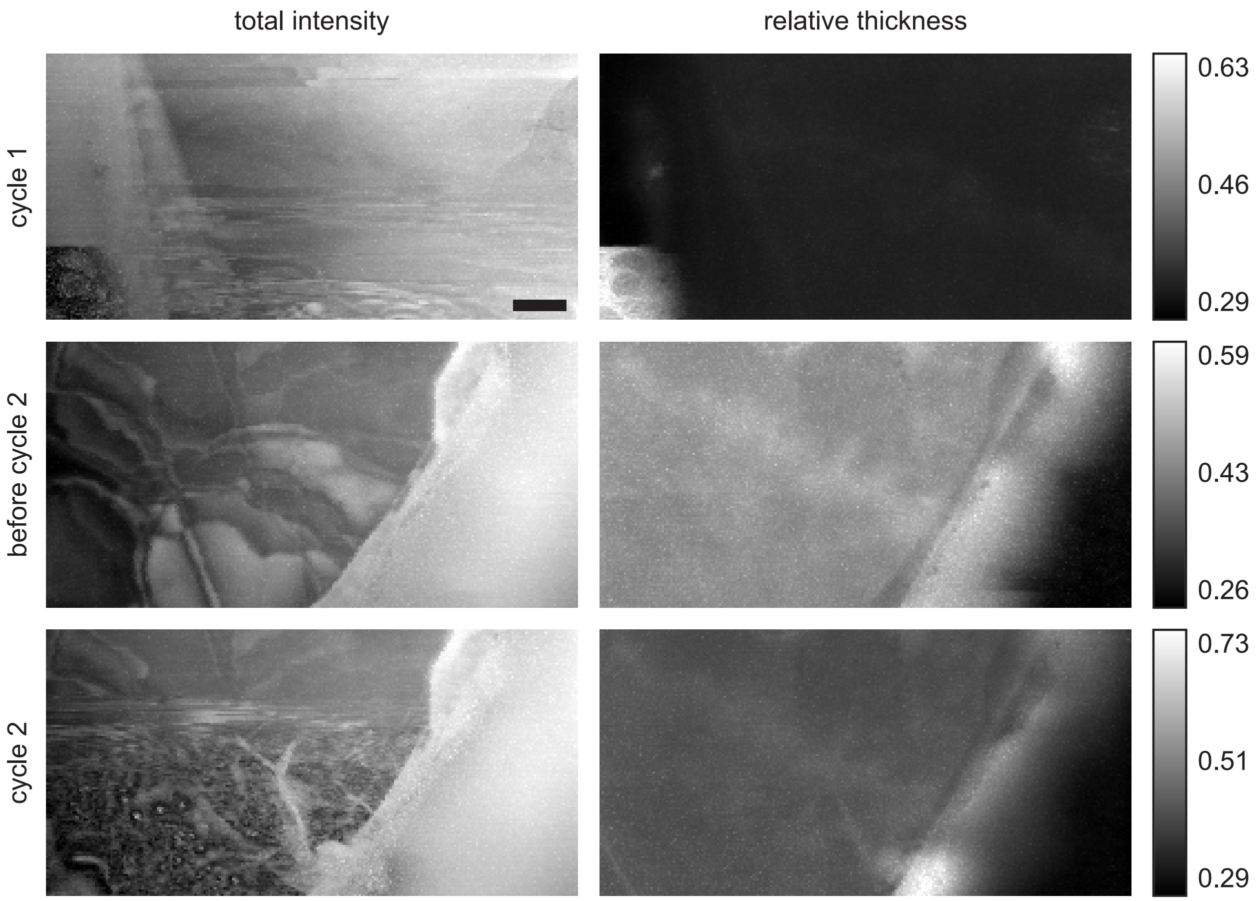

Figure S4: **EELS thickness maps, first sample.** These six images are constructed from the spectrum images used to generate Figs. 2–3. Created by integrating over the entire energy spectrum, the images in the left column give a 'bright field' representation of the sample, illustrating its structural evolution during cycle 1 and 2, and its (static) structure between the cycles. In the right column, the relative thickness maps are created using the 'log-ratio' method [50]. The intensity scale bars describe the dimensionless thickness ratio t/λ, where t is the sample thickness and λ is the electron mean free path (mfp). For the imaging conditions used, the following materials have an estimated [50] mfp, given in parentheses, of: lithium (200 nm), lithium hydride (220 nm), graphite (160 nm), Li_2CO_3 (160 nm), EC:DMC (180 nm), and silicon nitride (140 nm). These mfp numbers, especially those of the compounds, are to be considered rough, as they are derived assuming standard elemental densities. By themselves, the two 20-nm-thick Si_3N_4 membrane windows separating the fluid cell interior from the TEM vacuum represent a t/λ of 0.29, within 10% of the minimum total thickness measured. Taking a mfp of 200 nm to be characteristic of the other materials, we see that the non-window material varies from near zero to 70-, 60-, and 90-nm-thick for the cycle 1, before cycle 2, and cycle 2 spectrum images respectively. Interestingly, the cell thickness varies both in space and in time. For these spectrum images a $t/\lambda \sim 0.5$ is typical, which corresponds to 40 nm of Si_3N_4 and 30–50 nm of LIB material.

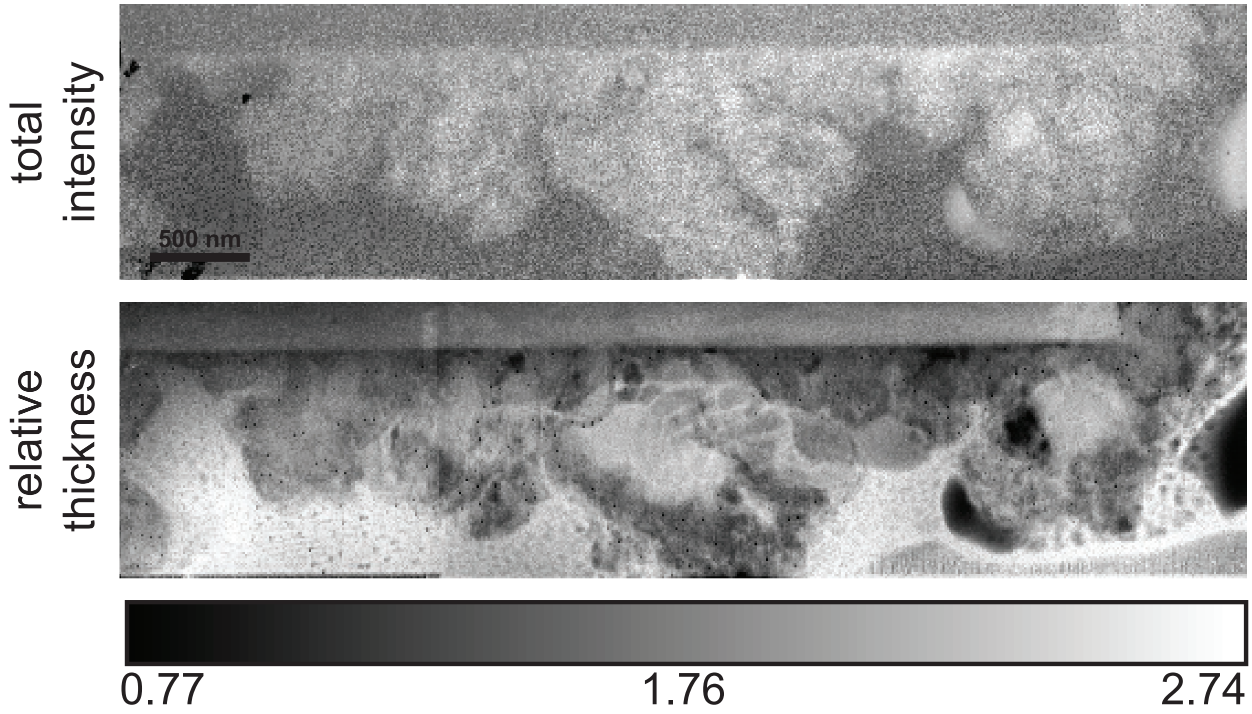

Figure S5: **EELS thickness map, second sample.** These two images are constructed from the spectrum image used to generate Fig. 4 using the methods of Fig. S4. This sample is thicker than the first: t/λ varies from 0.8 to 2.7, of which 0.5 to 2.4 can be attributed to the contents of the fluid cell. Again taking a mfp of 200 nm to be characteristic of the LIB materials, we see that the non-window material varies from 100 to 500-nm thick. Unlike the ADF STEM image (Fig. 4A), which captures electrons scattered to angles 20–40 mrad and highlights the Li and LiH dendrites within the SEI, the total intensity image highlights the SEI and dendrites uniformly. The relative thickness image shows the dendrites to have larger t/λ relative to the rest of the SEI.

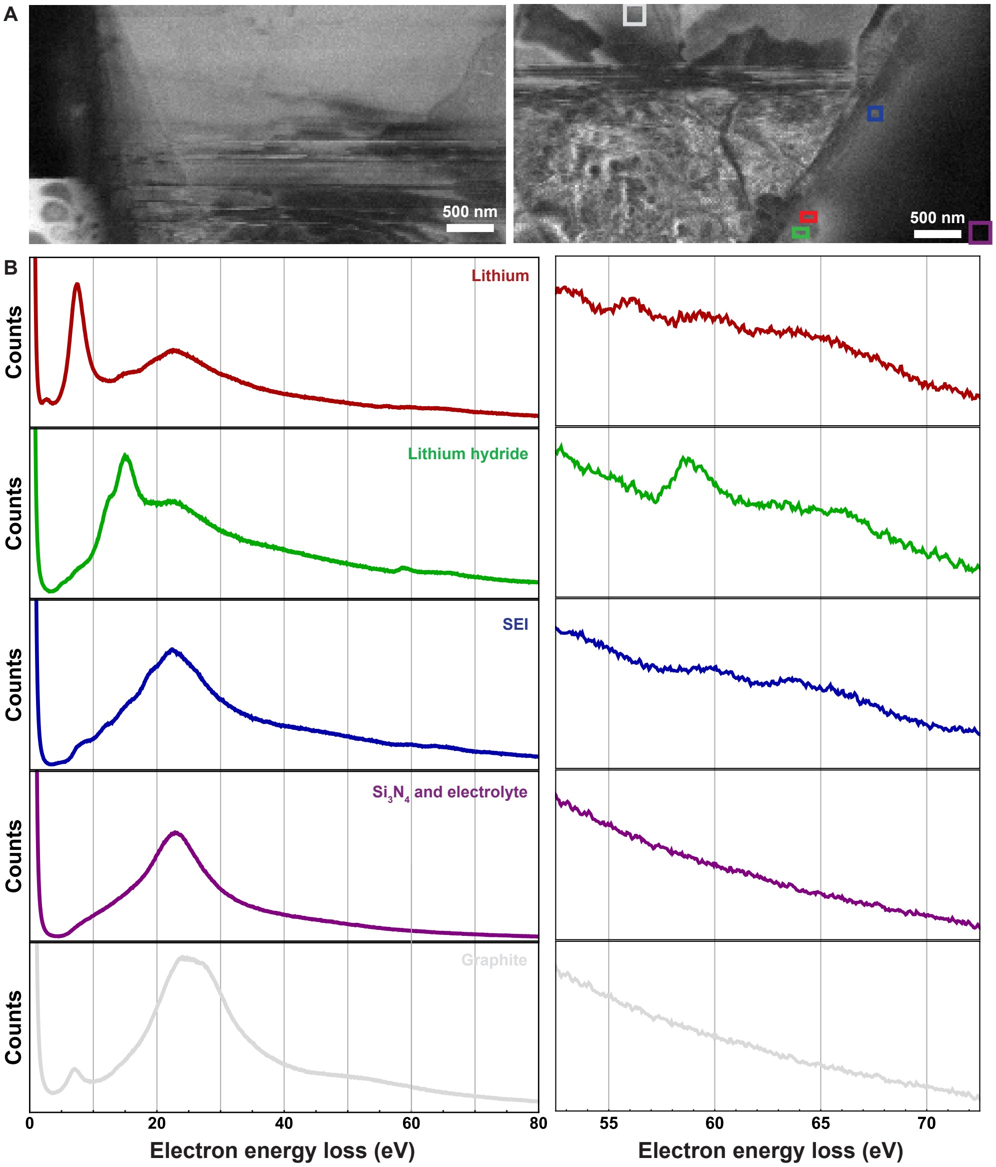

Figure S6: **Multiple linear least-squares (MLLS) fitting reference spectra for Fig. 2. (A)** The left and right ADF images show the data of Fig. 2B and Fig. 2E, respectively. Colored boxes in the right ADF image indicate regions that, due to their location and morphological and spectral uniformity, can be considered to be representative of a given material. **(B)** Representative spectra for Li, LiH, the SEI, the Si_3N_4 & electrolyte, and the graphite, as determined by averaging the acquired spectra across the regions framed by correspondingly colored rectangular boxes in part (A). No subtractions are performed. For instance, the 'graphite' spectrum also includes a contribution from the Si_3N_4 membrane window, which spans the entire field of view. MLLS fitting is applied over the energy range 4–40 eV. Li K-edge signals (right plots, which are zoom views of the left plots) show evidence of Li in the lithium, the lithium hydride, and the SEI, but not in the other materials, as expected.

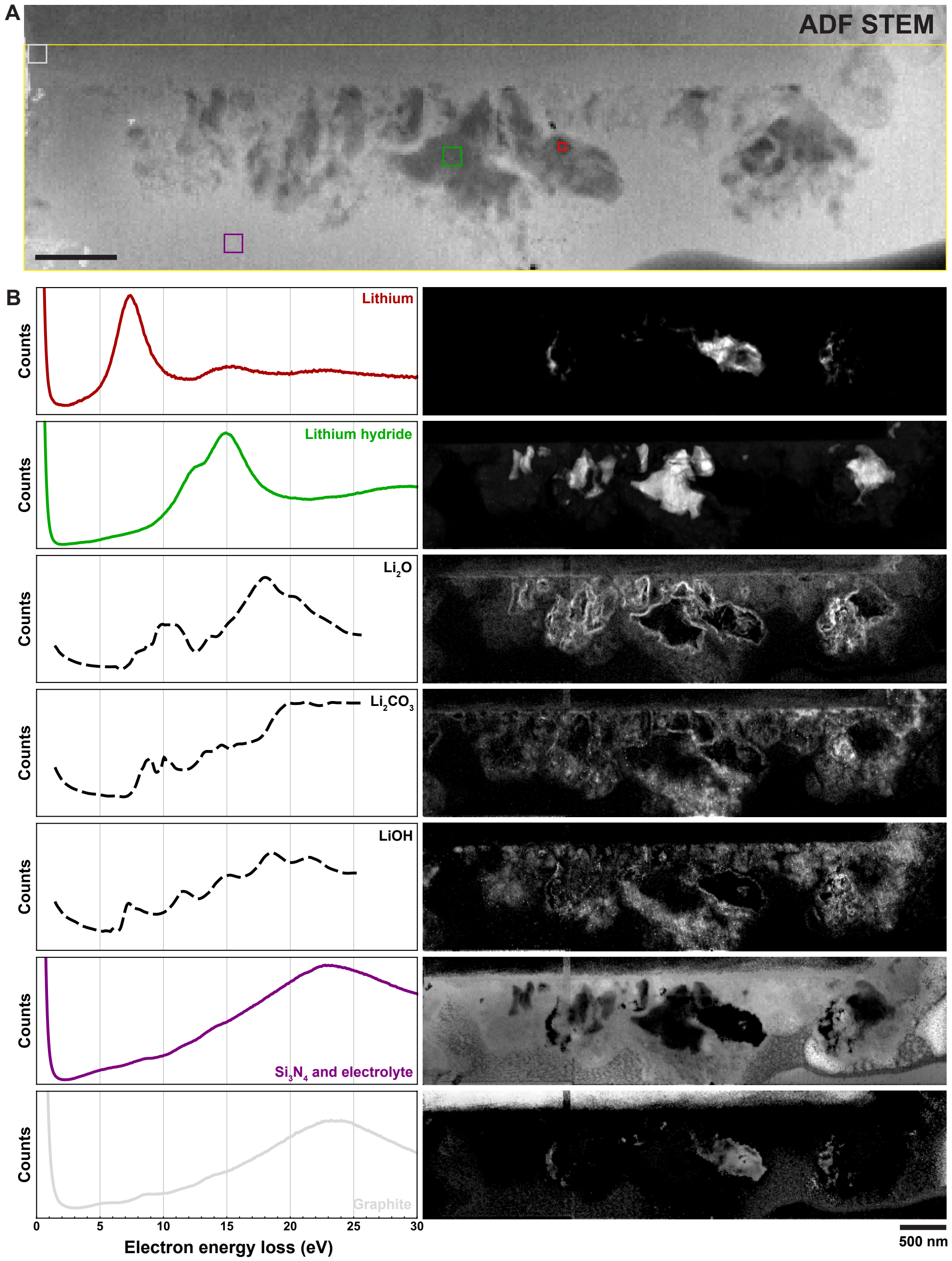

Figure S7: **Multiple linear least-squares (MLLS) fitting reference spectra and component maps for Fig. 4.** On the ADF survey image **(A)**, a reproduction of Fig. 4A, rectangular boxes indicate the spectrum image field of view (yellow) and the locations chosen to produce representative spectra **(B)** of Li (red), LiH (green), the Si_3N_4 membrane window and electrolyte (purple), and the graphite (gray). As described previously (Fig. S6), no subtractions are performed. Although the ZLP is shown in the spectra acquired in this spectrum image (the others are from [48]), only the window 5–25 eV is used for fitting.

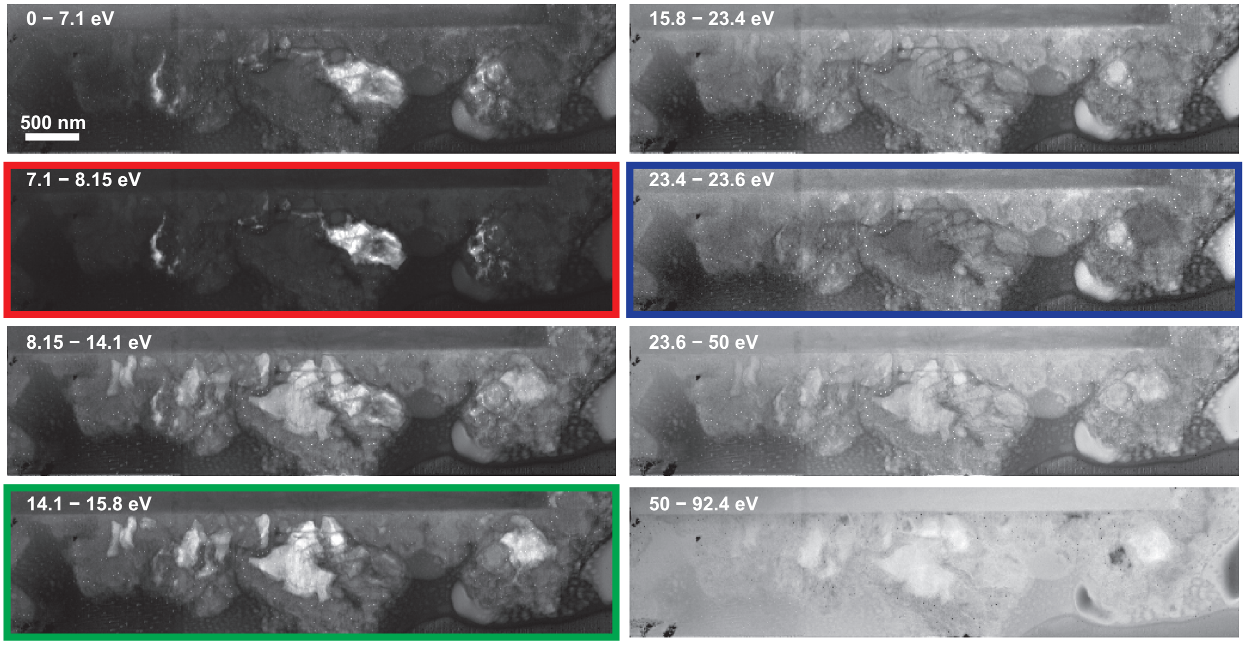

Figure S8: **Energy segmented spectrum image of a well developed SEI.** To give a still rendition of Movie S4, the source spectrum image is segmented into 8 different energy slices, each of which corresponds to the signal integrated over the indicated energy bandwidth. The bandwidths vary from slice to slice. The frames with the red, green, and blue colored outlines show energy windows that indicate roughly the spatial distribution of the Li, LiH, and the SEI, respectively. This crude segmented display corroborates the more sophisticated MLLS approach presented in Fig. 4 of the main text.

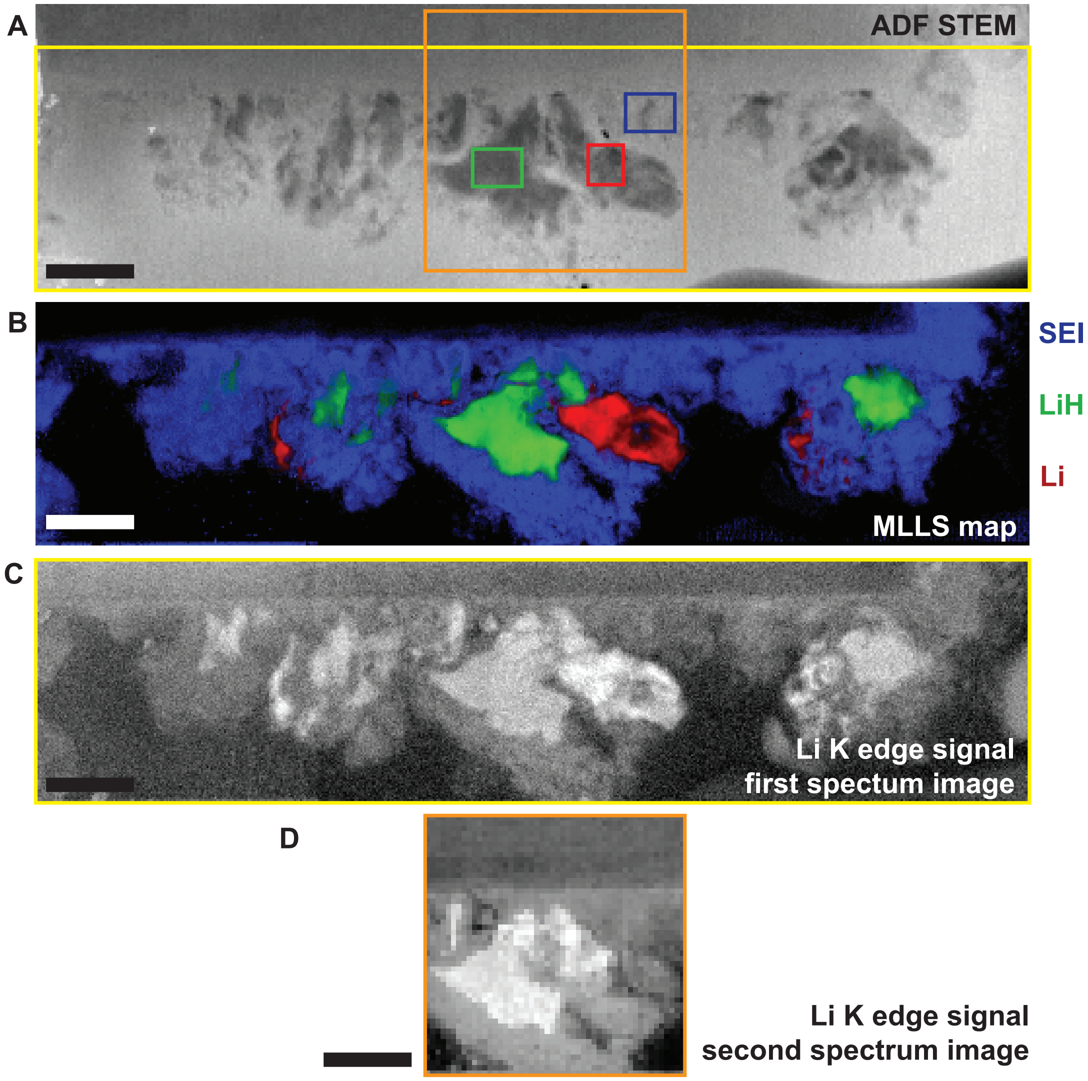

Figure S9: **Li spectrum imaging, map summary.** The ADF image **(A)**, a reproduction of Fig. 4A, shows the sample before any spectrum imaging. The orange box indicates the field of view of the second, 'core-loss' spectrum image of the same sample (Table S1). The MLLS map **(B)** shows the spatial distribution of the lithium, the lithium hydride, and the SEI, as determined by fits to the low-loss portion of the first spectrum image using reference spectra acquired from the indicated regions. At a given pixel, integrating the individual background-subtracted core-loss intensities provides an indication of the quantity of corresponding element at that location. Applying this algorithm, which is described in detail in the caption of Fig. S11, to the Li K-edge signal produces **(C)** and **(D)** from the first (102.4 eV bandwidth) and second (2048 eV bandwidth) spectrum images, respectively. [The map (D) is a reproduction of Fig. S11C.] Relative to the Li K-edge signal in the lithium hydride dendrite, the Li K-edge signal in the lithium dendrite goes from being more intense in (C) to less intense in (D). Thus the lithium appears to be less stable than the lithium hydride under these (room temperature liquid cell) imaging conditions. The scale bars are 500 nm.

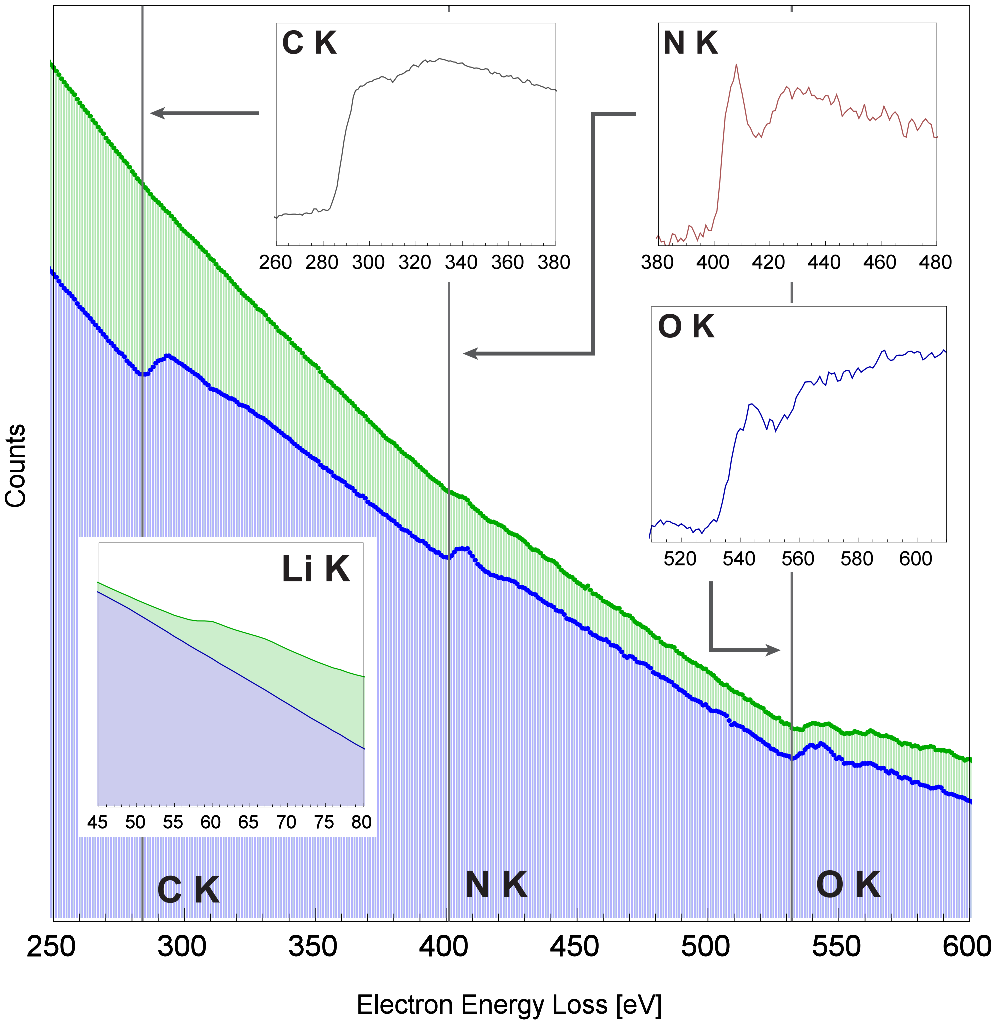

Figure S10: **2048 eV bandwidth spectrum image, spectra.** Average spectra collected from the correspondingly colored green and blue rectangles in the 11–2059 eV bandwidth spectrum image (Fig. S9). The insets show the Li K-edge (lower left), and the background-subtracted C, N, and O K-edges from the SEI (upper right), respectively, all from the same spectrum. K-edge resonances at 284 and 532 eV reveal the presence of carbon and oxygen, respectively, in the SEI. The nitrogen core-loss signal at 402 eV we attribute to the silicon nitride membrane windows. With this larger bandwidth the Li K-edge is still visible in the lithium hydride, but not in the SEI. The observed carbon and oxygen K-edge structure in the SEI is consistent with lithium carbonate and lithium semi-carbonates (i.e. alkyl carbonates) [8, 10, 30, 34], but the signal-to-noise ratio and the confounding effects of the electrolyte background together prevent the stoichiometry or chemical bonding states from being quantified precisely (Fig. S11).

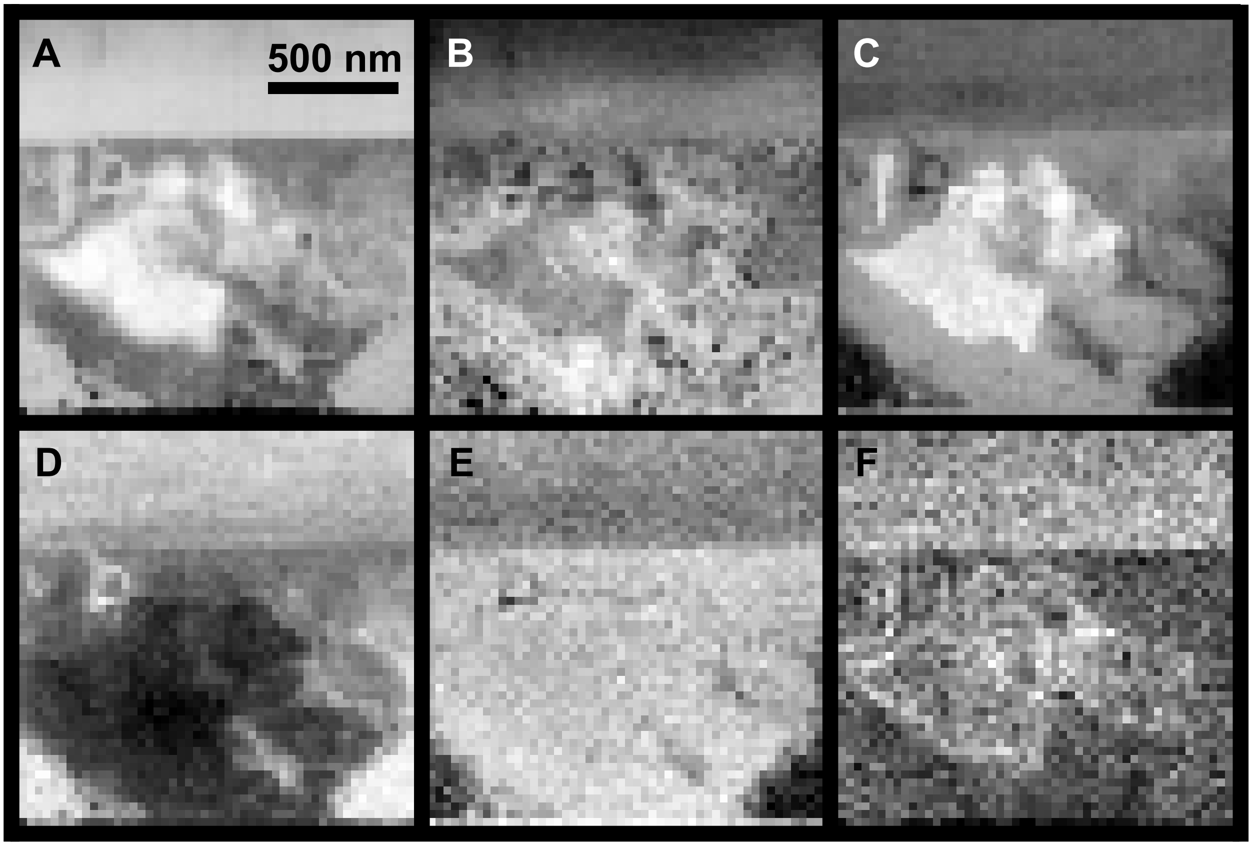

Figure S11: **2048 eV bandwidth spectrum image, maps.** **(A)** Total intensity/virtual bright field formed by integrating the intensity over the entire 2048 eV bandwidth of the 'core-loss' spectrum image (Fig. S9, Table S1). **(B)** Annular dark field image acquired simultaneously. **(C)** Lithium signal after the Li K-edge at 55 eV, **(D)** carbon signal after the C K-edge at 284 eV, **(E)** nitrogen signal after the N K-edge at 402 eV, and **(F)** oxygen signal after the O K-edge at 532 eV. To extract the Li core-loss signal (C) we subtract an exponential background determined in a 20 eV bandwidth immediately below the edge, and integrate the remaining signal in a 11 eV bandwidth after the edge. To extract the C, N, and O core-loss signals (D,E,F), we subtract a power-law background determined in a 70 eV bandwidth 5–10 eV before the corresponding edge, and integrate the remaining signal in a 105 eV bandwidth after the edge. The lithium hydride dendrites show a significantly stronger Li signal than the lithium metal and some indication of an oxide shell [30]. The graphite is clearly visible at the top of all four (unnormalized) K-edge maps. Its presence in the nitrogen map, which might otherwise be expected to show a flat field, indicates that the K-edge intensities cannot be simply interpreted as proportional to the number of atoms of the corresponding element present.

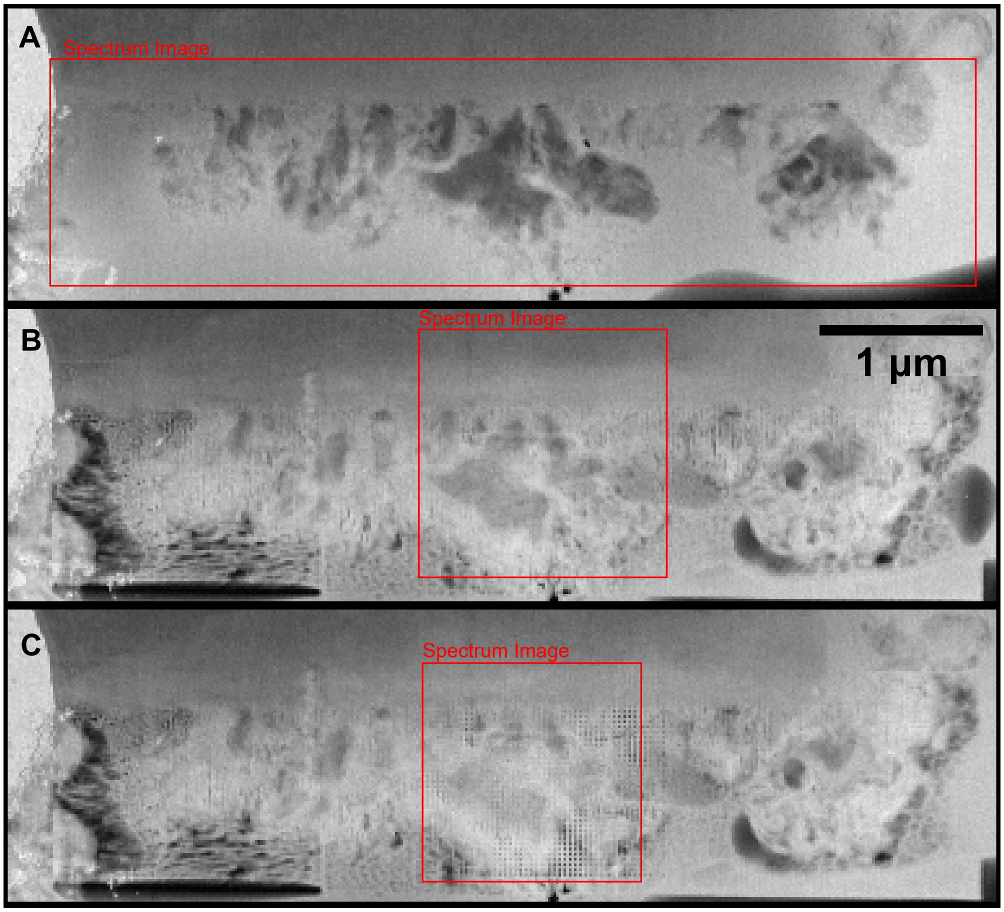

Figure S12: **Dose effects in survey images.** Survey images acquired *before* the **(A)** first, **(B)** second, and **(C)** third spectrum images of the Fig. 4 sample. Acquiring the first spectrum image damages the pristine SEI (A), as evidenced by the decreased contrast difference between the Li and LiH dendrites and their surroundings (B). The second spectrum image, with its 10× longer dwell times (Table S1), imprints a visible grid of damage (C) in the SEI, but not elsewhere. Because the SEI is so obviously compromised after the second spectrum image, no data from the third spectrum image are presented in this paper.

Improving the signal-to-noise ratio for spectroscopy of the SEI represents a major challenge, as the carbon-containing components of the SEI are more beam sensitive than the other cell components (i.e. the Li, the LiH, the graphite electrode, and the electrolyte) [31,33]. This sensitivity is evident in the first spectrum image (Movie S4), which shows single pixel spots that are noticeably brighter than their surroundings below 40 eV. We attribute these spots to chance overlap of the beam with a region previously damaged during survey imaging. They appear only in the SEI, and become more common with repeated imaging. Spectrum imaging, with its characteristically longer dwell times, makes the damage obvious even to ADF imaging by imprinting an array in the SEI that reflects the mismatch between the probe size and the pixel size (Table. S1). We conclude that, in its native, room-temperature liquid electrolyte, even comparatively low-dose survey imaging damages the SEI, and that the ability of any spectrum to faithfully reflect the SEI's constituent compounds in the unimaged, pristine state must be critically considered in light of the total dose delivered.

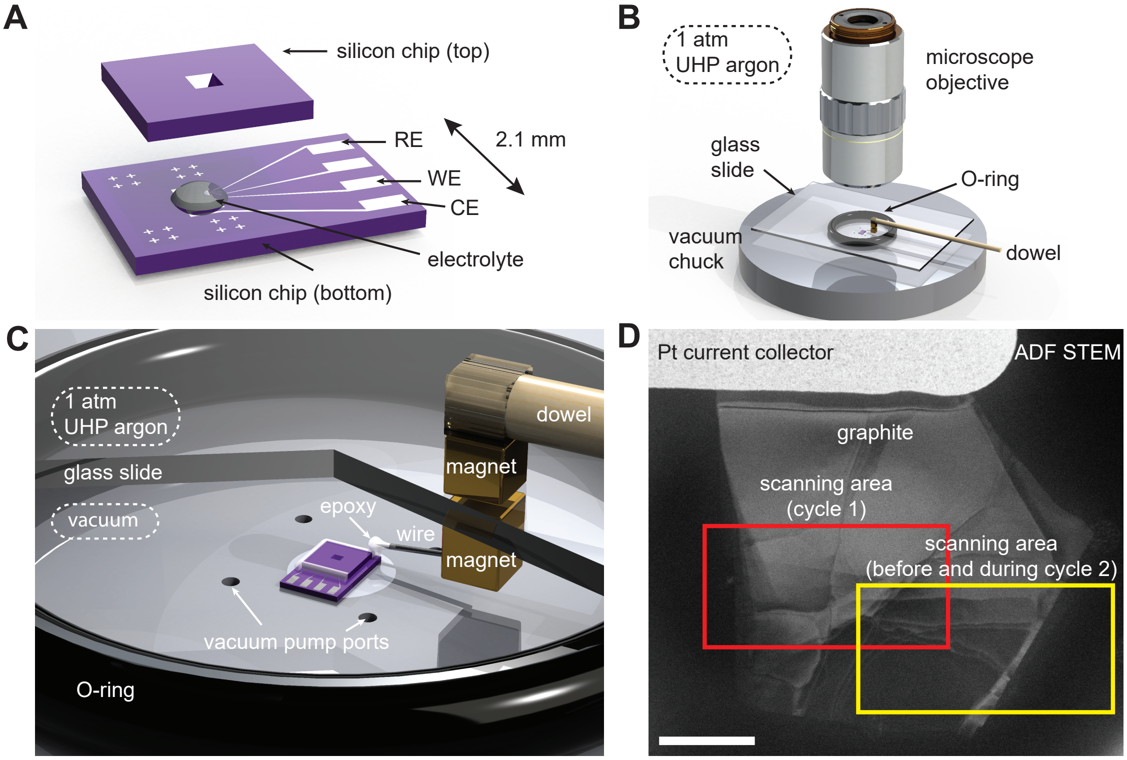

Figure S13: **(Fig. 1, vector format) Liquid cell imaging platform for STEM EELS. (A)** Schematic showing an exploded view of the fluid cell. A droplet of 1M $LiClO_4$ in EC:DMC serves as the electrolyte and as the lithium source (Fig. S2). The top and bottom chips are epoxied together on three sides in a glove bag under 1 atm of ultra-high-purity argon. **(B)** The fourth and final edge is sealed inside a vacuum chamber assembled inside the glove bag (not shown). **(C)** A zoom view with a cutaway in the glass slide shows how opposing NdFeB magnets provide mechanical access for epoxying inside the vacuum chamber (Fig. S1). **(D)** ADF STEM image of a pristine graphite flake contacted by a platinum electrode within a sealed fluid cell. Red and yellow boxes outline the scan areas for the first and second lithiations, respectively. The scale bar is 2 μm.

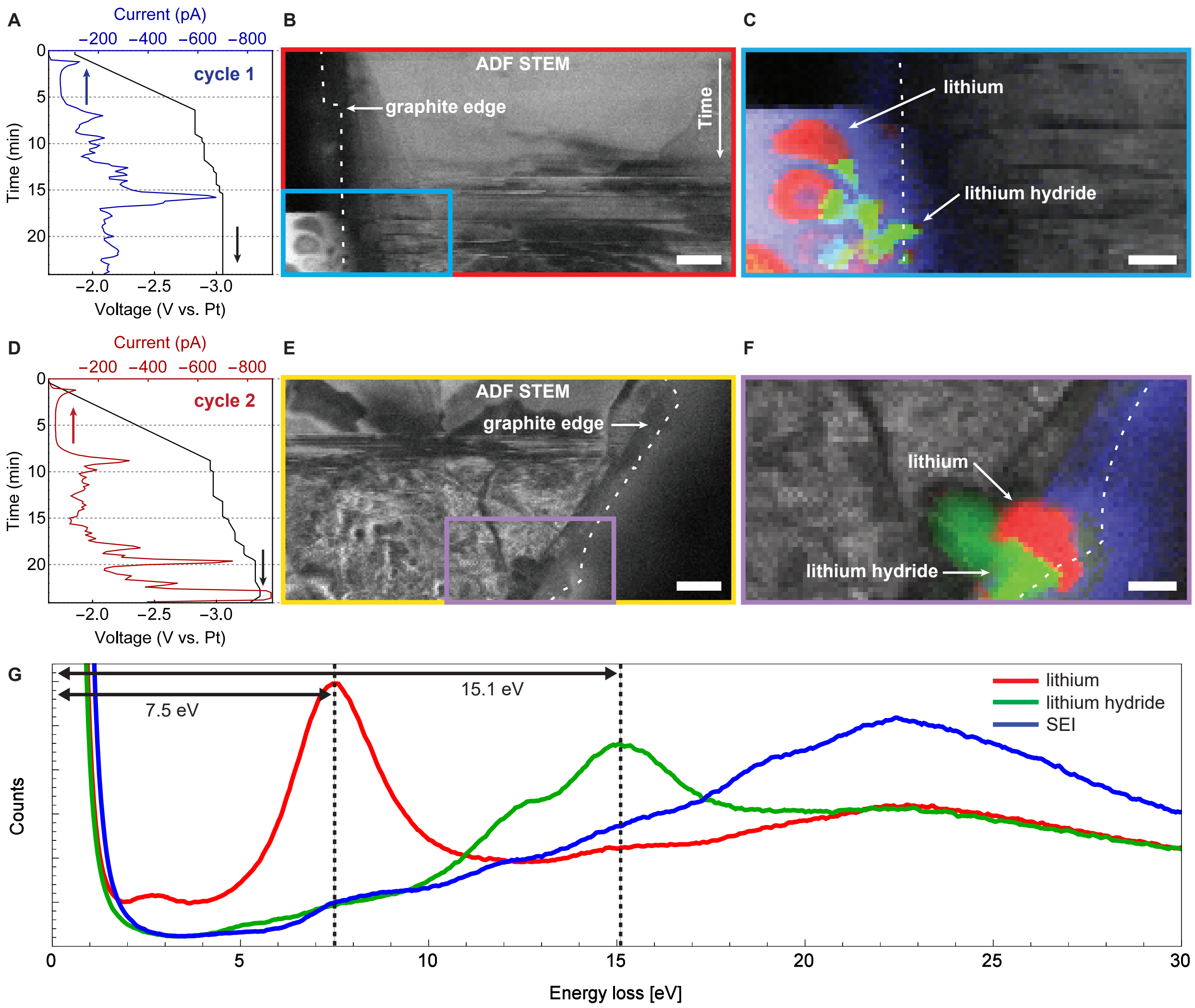

Figure S14: **(Fig. 2, vector format) Lithiating a single crystal graphite flake.** White dashed lines indicate the graphite's true edge (Fig. S3). The measured current data from the first lithiation **(A)** is aligned and averaged so that each time point represents a single row of pixels in **(B)** the simultaneously-acquired ADF STEM image. **(C)** A zoom view of the region indicated in (B), with red, green, and blue overlays on the ADF STEM image that indicate the presence of Li, LiH, and SEI, respectively, as determined by MLLS fitting (Fig. S6). **(D–F)** The corresponding data for lithiation 2. **(G)** Low-loss EELS spectra of lithium metal (red), lithium hydride (green), and the solid-electrolyte interphase (blue). The scale bars are 500 nm in (B, E) and 200 nm in (C, F).

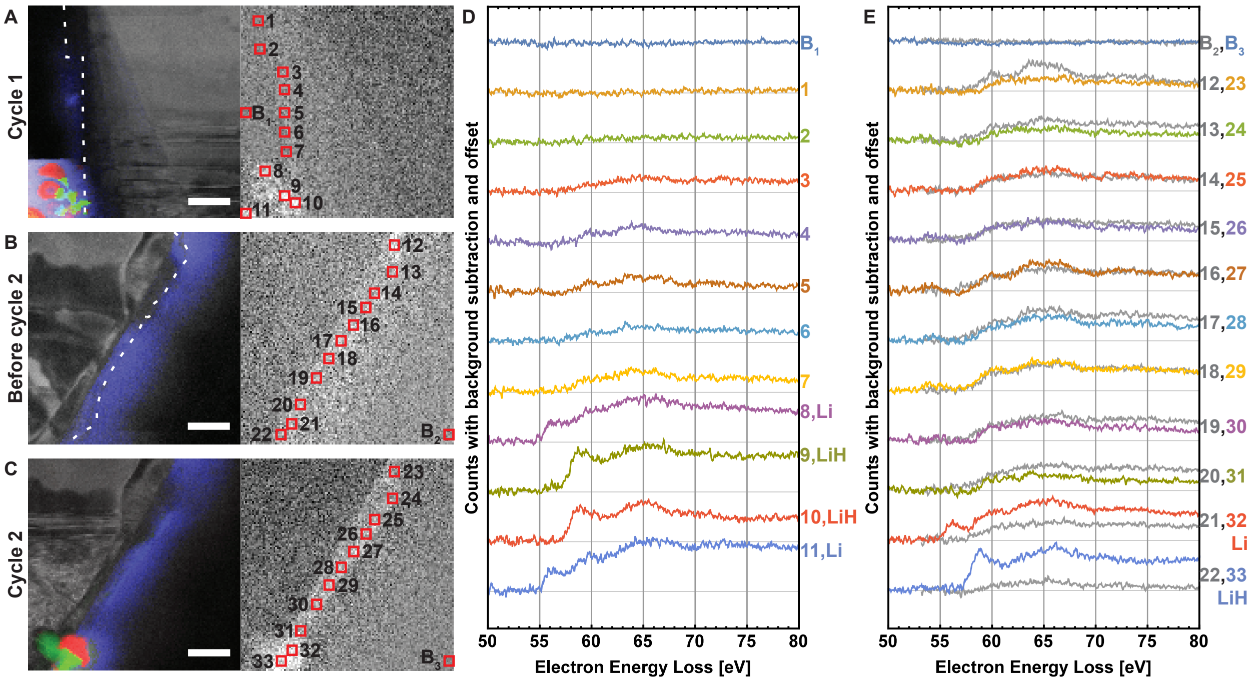

Figure S15: **(Fig. 3, vector format) Chemical evolution at the solid-liquid interface during lithiation.** ADF STEM images of the graphite flake with MLLS color overlays (see Fig. 2) **(A)** during the first lithiation, **(B)** before cycle 2, and **(C)** during lithiation 2 (left) and the corresponding, energy-filtered maps of the EELS intensity integrated over 50–80 eV, after background subtraction (right). We emphasize that, once the reference spectra are chosen, the MLLS mapping is entirely automated over the whole field of view. **(D, E)** Background-subtracted spectra summed over the regions indicated by red boxes in (A–C). Each box is 5×5 pixels, which corresponds to 107 nm$\times$107 nm. The scale bar is 500 nm in (A–C).

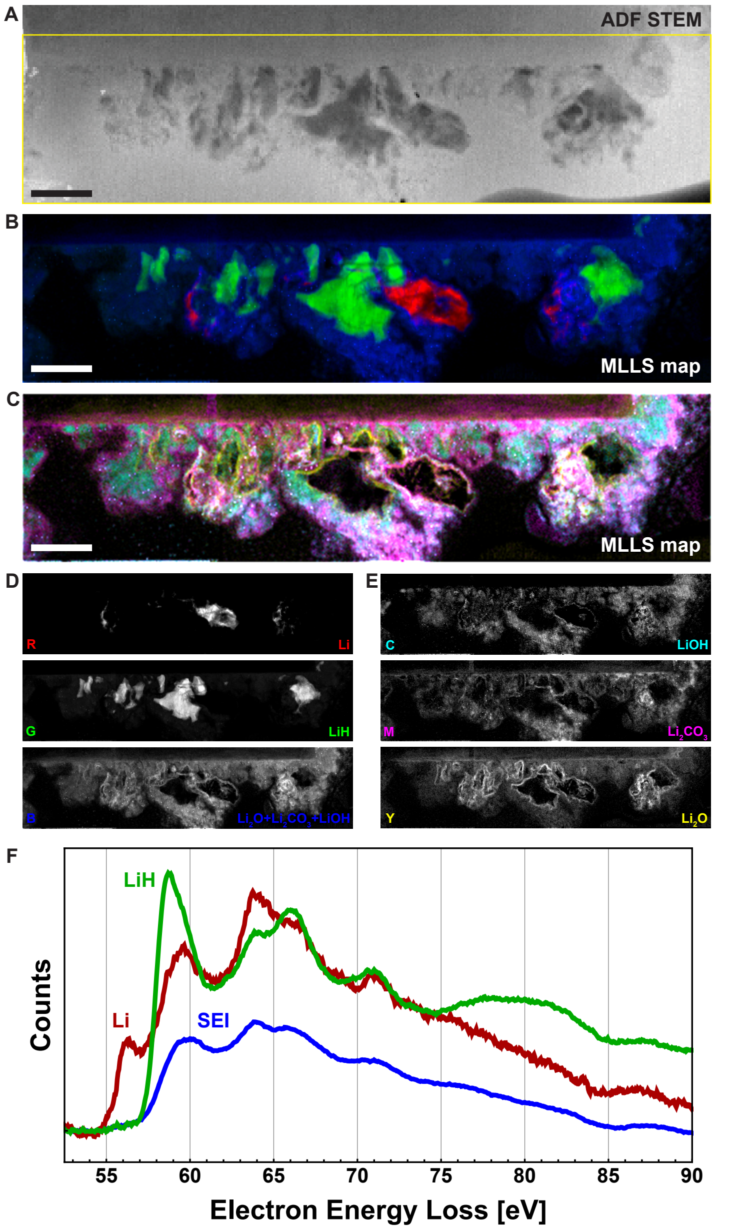

Figure S16: **(Fig. 4, vector format) Denser, more developed SEI. (A)** ADF STEM survey image of a graphite electrode with an SEI formed by two complete lithiation-delithiation cycles *in situ*. The graphite occupies a rectangular region extending from the upper left corner across 90% of the top edge. **(B)** Red, green, and blue (RGB) and **(C)** cyan, magenta, yellow (CMY) MLLS composite images (see Methods) based on a 102.4 eV bandwidth spectrum image with field of view indicated by the yellow box in (A). **(D, E)** Corresponding grayscale MLLS images showing the individual components of (B, C), respectively (Fig. S7). The scale bars are 500 nm. **(F)** Background-subtracted Li core-loss signals found by calculating intensity-weighted averages over the maps (D).
